# Discovery of Mycothiogranaticins from Streptomyces vietnamensis GIMV4.0001 and the Regulatory Effect of Mycothiol on the Granaticin Biosynthesis

**DOI:** 10.3389/fchem.2021.802279

**Published:** 2021-12-23

**Authors:** Ming-Rong Deng, Yan Li, Xiao Luo, Xiang-Ling Zheng, Yuchan Chen, Yu-Lian Zhang, Weimin Zhang, Hao Zhou, Honghui Zhu

**Affiliations:** ^1^ Key Laboratory of Agricultural Microbiomics and Precision Application — Ministry of Agriculture and Rural Affairs, Guangdong Provincial Key Laboratory of Microbial Culture Collection and Application, State Key Laboratory of Applied Microbiology Southern China, Guangdong Microbial Culture Collection Center (GDMCC), Institute of Microbiology, Guangdong Academy of Sciences, Guangzhou, China; ^2^ Key Laboratory of Functional Molecules Analysis and Biotransformation of Universities in Yunnan Province, School of Chemical Science and Technology, Yunnan University, Kunming, China

**Keywords:** mycothiol, MshA, MST, regulation, *Streptomyces vietnamensis*, granaticin, actinomycete, sulfur-containing polyketide

## Abstract

Granaticins are benzoisochromanequinone polyketides with remarkable antibacterial and anticancer activities. Three sulfur-containing granaticin congeners, mycothiogranaticins A (**1**), B (**2**) and granaticin MA (**3**) were discovered from a granaticin-producing strain of *Streptomyces vietnamensis* GIMV4.0001. Two of them were structurally determined with mycothiol or N-acetylcysteine moieties and found to be bio-actively reluctant. Disruption of the *mshA* gene (*SVTN_RS20640*) that encodes the D-inositol-3-phosphate glycosyltransferase crucial for mycothiol biosynthesis, fully abolished the production of mycothiogranaticins. The result substantiated that the newly discovered mycothiogranaticins are consequences of the combination of the granaticin and mycothiol biosynthetic pathways. The overall granaticin production of the Δ*mshA* mutant strain was unexpectedly decreased by at least more than 50%, while similar production level of granaticins to that of the wild type strain was observed in an mycothiol-S transferase gene (*SVTN_RS22215*) disruptant Δ*mst*. These results indicated that the mycothiol deficiency was responsible for the decreased production of granaticins. Mycothiol may positively regulate the biosynthesis of granaticin possibly by maintaining the cellular redox balance. To the best of our knowledge, this is the first report that mycothiol can not only be a direct building block of polyketides but also play a regulatory role in the polyketide biosynthesis.

## Introduction

Bacterial aromatic polyketides are pharmaceutically important natural products with remarkable structural diversity, some of which have been developed and most commonly used as antibiotics and anticancer drugs, such as oxytetracycline, tetracenomycin, doxorubicin and aclacinomycin. Therefore, deeper explorations of the structural diversity of bacterial aromatic polyketides would enable this class of compounds as continuous sources for drug development. Sulfur incorporation can significantly expand the structural diversity and bioactivities of many naturally occurring molecules (Hai et al., 2021), many sulfur-containing natural products, such as penicillin and ixabepilone, have been approved for clinic therapy. However, bacterial sulfur-containing aromatic polyketides are relatively rare, with only a few examples reported over the past decades (Etoh et al., 1987; Ohta et al., 1987; Rohr and Zeeck, 1987; Miyata et al., 1992; Aoyama et al., 1993; Carney et al., 1997; Kulanthaivel et al., 1999; Sasaki et al., 2010; Taguchi et al., 2013; Wang et al., 2013; Woo et al., 2013; Taguchi et al., 2015; Bilyk et al., 2016; Che et al., 2016; Xie et al., 2016; Nakashima et al., 2017; Matsuo et al., 2019a; Bae et al., 2019; Matsuo et al., 2019b; He et al., 2019; Fang et al., 2020; Cao et al., 2021). Amongst these reports, naquihexcin A could inhibit the proliferation of the adriamycin resistant human breast cancer (Che et al., 2016); nanaomycin K showed an inhibitory effect on the epithelial-mesenchymal transition (Matsuo et al., 2019a); and naquihexcin E exhibited a notable anti-HIV activity (He et al., 2019). These suggested promising potentials of these privileged sulfur-containing polyketides for drug development.

**FIGURE 1:**
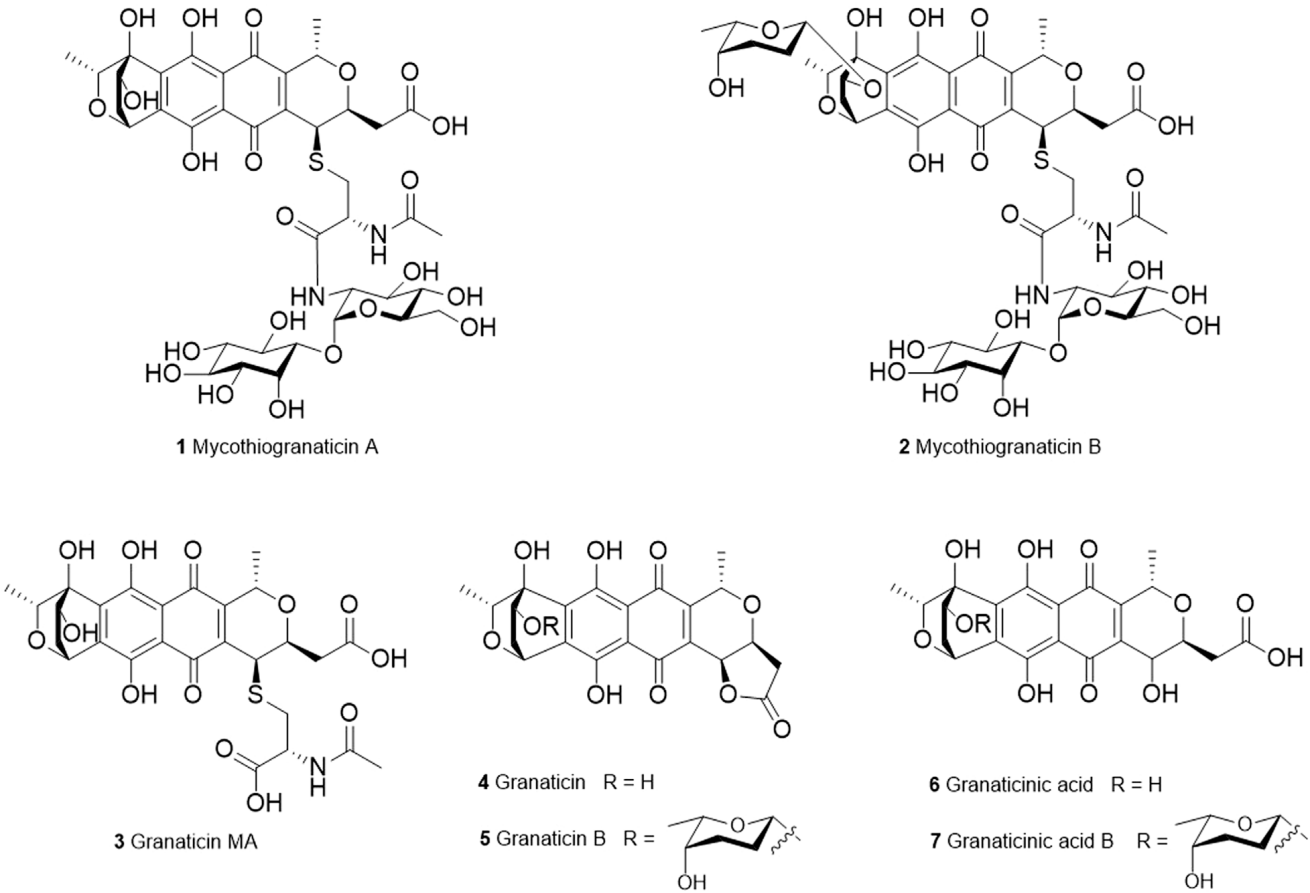
Structures of granaticins and mycothiogranaticins.

Granaticins are members of the benzoisochromanequinone (BIQ) polyketides (Figure 1), showing a wide range of biological activities on both microbes and higher organisms. They are highly active against Gram-positive bacteria and protozoa and exhibit cytotoxicity against many cancer cell lines *in vitro* at nM to µM levels as well as P-388 lymphocytic leukemia in mice (Corbaz et al., 1957; Chang et al., 1975; Elson et al., 1988). Granaticins inhibit bacteria by interfering with tRNA^Leu^ aminoacylation process resulting in failure to synthesize proteins and RNAs (Ogilvie et al., 1975a; b). The cytotoxicity was reported initially to be attributed to the inhibition of ribosomal RNA maturation (Heinstein, 1982). More recently, granaticins were found to specifically inhibit farnesyltransferase (Iwasaki and Omura, 2007), inosine 5′-monophosphate dehydrogenase (Ren et al., 2008) and cell division cycle 7 kinase (Frattini et al., 2011), which are important targets for the development of anticancer drugs (Ratcliffe, 2006; Agrawal and Somani, 2009; Swords et al., 2010). Therefore, granaticins are the highest ranked BIQ members with clinical potentials.

*Streptomyces vietnamensis* is a recently designated species that can produce granaticins (Zhu et al., 2007; Deng et al., 2011a). The metabolites from the granaticin pathway of this strain were systematically characterized (Deng et al., 2020). Here we report the discovery of three sulfur-containing granaticin congeners, mycothiogranaticins A (**1**), B (**2**) and granaticin MA (**3**), from *S. vietnamensis* GIMV4.0001, and the involvement of mycothiol in the granaticin biosynthesis, serving as both a structural building block and a biosynthetic regulator.

## Materials and Methods

### Strains, Plasmids, Biochemicals and Growth Conditions

Strains, plasmids, and polymerase chain reaction (PCR) primers used in this study were listed in Supplementary Table S1, S2, respectively. *Escherichia coli* NEB Turbo was used for general cloning and plasmid preparation. *E. coli* ET12567/pUZ8002 (MacNeil et al., 1992) was used as the donor host for intergeneric conjugation. *S. vietnamensis* GIMV4.0001 (Zhu et al., 2007) is a wild-type granaticin (**4**) producer. The temperature-sensitive plasmid pKC1139 (Bierman et al., 1992) was used for generating the in-frame disruption plasmids pKC-Δ*mshA* and pKC-Δ*mst*. The integrative plasmid pSET-KasO* (Pan et al., 2017) was used for the gene complementation purpose. PCR primers were ordered from and synthesized by GENEWIZ. Phanta Max Super-Fidelity DNA polymerase and ClonExpress MultiS One Step Cloning Kit were purchased from Vazyme Biotech Co., Ltd, China, and the reactions were performed according to the manufacturer’s procedures. Kits for gel extraction and plasmid preparation were products of Magen Co., Ltd (China). Other common biochemicals and medium components were purchased from standard commercial sources. DNA sequencing was performed by GENEWIZ. *E. coli* strains containing plasmids were cultured in LB medium at 37 °C, with shaking at 200 rpm, supplemented with appropriate antibiotics as required. *Streptomyces* strains were grown at 28 °C on ISP2 agar medium for sporulation or in liquid YEME medium (0.3% yeast extract, 0.5% tryptone, 0.3% malt extract, 1% glucose, 5 mM MgCl_2_) for growth of mycelium, isolation of total DNA and granaticin (**4**) production. The liquid Gauze’s synthetic medium No.1 was also used for parallel analysis of granaticin (**4**) production. *E. coli*-*Streptomyces* conjugations were performed on IPS4 agar medium.

### Fermentation, Isolation and Structural Elucidation

The seed cultures were prepared from 2-days fermentation broths rotationally incubated at 220 rpm and 28 °C in the liquid YEME medium. For large-scale fermentation (20 L), fifty 2000-mL baffled flasks containing 400 mL of liquid YEME with 5% (in volume) seed culture were incubated under the foresaid conditions for 6 days. At the end of the fermentation, 5% (w/v) resins (Amberlite^®^ XAD16, Shanghai Macklin Biochemical Co., Ltd, China) were added into the cultures, and a 3-h extended incubation with rotation was applied to allow the metabolites to be absorbed into the resins. Then the resins were harvested, cleaned and air-dried. Methanol was used for extraction. The organic extracts were concentrated *in vacuo*. The crude extract was fractionated on a silica gel column and eluted with a stepwise gradient of CH_2_Cl_2_−CH_3_OH (100:0, 98:2, 96:4, 94:6, 92:8, 9:1, 8:2, 7:3, 1:1, 0:100). The elution volume was 200 mL for each gradient. The eluents were analysed by TLC and then combined. The combined fractions were further analysed by LC-MS. The fractions that contain the interested *m/z* values were further chromatographed over a preparative reversed-phase HPLC column (Waters XBridge^®^ Prep C18 10 µm OBD, 19 × 250 mm) with a gradient elution (13 mL/min) of CH_3_OH in H_2_O containing 0.1% formic acid. The targeted subfractions were then passed through several rounds of semipreparative HPLC (Agilent ZORBAX Stable Bond 80Å Phenyl Column, 9.4 × 250 mm, 5 µm) to obtain pure compounds. Compounds **1** and **3** were purified with retention times of 10.6 and 17 min, respectively, using an isocratic elution (2.5 mL/min) of 55% CH_3_OH in H_2_O containing 0.1% formic acid. The retention time of compound **2** was 11.2 min when using a 15-min gradient elution (3 mL/min) from 60–100% CH_3_OH in H_2_O containing 0.1% formic acid. The ^1^H and ^13^C NMR spectra were done on either a Bruker Avance III HD 600 MHz or Avance III Ultrashield 700 MHz with QCI Cryoprobe spectrometer at 298 K. Optical rotations were measured with an Anton-Paar’s MCP500 polarimeter. The experimental CD spectrum of compound **1** was collected in methanol at a concentration of 0.5 mg/mL on an Applied Photophysics Chirascan spectrometer using a quartz cell with path length of 10 mm. The ECD calculation was performed by using the density functional theory (DFT) with the Gaussian 09 package (Frisch et al., 2009). The preliminary conformational distributions search was performed *via* molecular mechanics using the MM+ method implemented in CONFLEX version 8.0 software. The obtained conformers were optimized at the B3LYP/6-31G level using Gaussian 09 software to give the energy-minimized conformers. Methanol was used as a solvent with the polarizable continuum model (PCM). Then, the optimized conformers were subjected to the calculations of ECD spectra using TDDFT at the B3LYP/6-31G (d, p) level. The overall calculated ECD curves were weighted by Boltzmann distribution (with a half-bandwidth of 0.35 eV) with a UV correction of 15 nm. The calculated ECD spectra were produced by SpecDis 1.64 software (Bruhn et al., 2013). The structures and absolute configurations were elucidated on the basis of extensive spectroscopic analyses including UV, MS, NMR and ECD spectra, and together with consideration of their biogenetic origins.

### Antibacterial and Cytotoxic Activity Assays

The minimum inhibitory concentrations (MICs) were determined using a 96-well plate format with Müller-Hinton (MH) broth (Wiegand et al., 2008). Cells of each strain at log-phase growth stage were adjusted to an OD_600_ = 0.5, then 100-fold diluted with MH broth. The diluted cell broth was pipetted with a volume of 98 μL into each well. Two microliters of each compound, serially diluted in DMSO, were added to each well. DMSO was used as negative control, and vancomycin and granaticin as positive controls. The MIC values were determined after incubation for 18 h either at 37 °C for *Staphylococcus aureus* or at 30 °C for *Micrococcus luteus*. Each MIC determination was performed in triplicate. A sulforhodamine B (SRB) colorimetric assay (Vichai and Kirtikara, 2006) was used to assess the potential cytotoxicity against the SF-268, MCF-7, HepG-2 and A549 cell lines. Cells (180 μL) with a density of 3 × 10^4^ cells/mL were seeded onto 96-well plates and incubated for 24 h at 37 °C, 5% CO_2_. Then 20 μL of compounds with various concentrations were added into the wells. Plates were further incubated for 72 h. After incubation, cell monolayers were fixed with 50% (wt/v) trichloroacetic acid (50 μL) and stained for 30 min with 0.4% (wt/v) SRB dissolved in 1% acetic acid. Unbound dye was removed by washing repeatedly with 1% acetic acid. The protein-bound dye was dissolved in 10 mM Tris base solution (200 µL) for OD determination at 570 nm using a microplate reader. Cisplatin was used as a positive control. Granaticin was also assessed for comparison purpose. All data were obtained in triplicate and are presented as means ± S.D. IC_50_ values were calculated with the SigmaPlot 14.0 software using a non-linear curve-fitting method.

### Chemical Analysis and Assessment of Biomass and Overall Production of Granaticins

LC-MS analysis was carried out, with an ESI source in negative ion mode, on an Agilent 6230 TOF mass spectrometry coupled to 1290 Infinity LC System equipped with an Agilent ZORBAX SB-C18 column (1.8 μm, 3.0 × 5.0 mm). Liquid chromatography for LC-MS analysis was performed using a 20 min solvent gradient (0.25 mL/min) from 10–100% CH_3_OH in H_2_O containing 0.1% formic acid. Yields of the four main products, granaticin (**4**), granaticin B (**5**), granaticinic acid (**6**) and granaticinic acid B (**7**) were used for assessment of the overall production of granaticins. Standard calibration curves for each product were generated by using the pure compounds (Supplementary Figure S1). The biomass was measured by using a volume of 20 mL of fermentation broth for each. All the tests were done in triplicate.

### Genetic Manipulation of *S. vietnamensis*

The *mshA* gene encodes the D-inositol-3-phosphate glycosyltransferase catalyzing the first step of mycothiol biosynthesis, and the *mst* gene dictates the mycothiol-S transferase catalyzing the transfer of mycothiol to various substrates (Newton et al., 2003; Newton et al., 2011). The protein sequences of *mshA* (*SCO4204*) from *S. coelicolor* A3 (2) (Park et al., 2006) and *Rv0443* from *Mycobacterium tuberculosis* H37Rv were used to BLAST against the genome sequence of *S*. *vietnamensis* GIMV4.0001 (Deng et al., 2015). The *SVTN_RS20640* gene, encoding a protein whose sequence shares an identity of nearly 80% with SCO4204, was preferentially considered as the *mshA* gene in *S*. *vietnamensis* (Supplementary Figure S2). The *SVTN_RS22215* gene, whose product was predicted to belong to the DinB superfamily, was the only candidate *mst* gene sharing 52.8% identity to Rv0443 (Supplementary Figure S3).

To construct the in-frame deletion mutant Δ*mshA*, two 1.7-kb DNA fragments flanking the *mshA* gene (*SVTN_RS20640*) were amplified using the primer pairs Sv-MshALF/R and Sv-MshARF/R. The plasmid pKC1139 was double digested with *Hin*dIII and *Eco*RI. The linearized plasmid together with the two PCR-amplified homologous arms were assembled by using a ClonExpress^®^ MultiS One Step Cloning Kit, affording the disruption plasmid pKC-Δ*mshA*. The recombinant plasmid pKC-Δ*mshA* was then subjected to sequencing to ensure that no mutations were introduced during the construction process. After passing through the non-methylating *E. coli* ET12567/pUZ8002, pKC-Δ*mshA* was introduced into *S*. *vietnamensis* GIMV4.0001 by intergeneric conjugation, following the established procedure (Deng et al., 2011b). Exconjugants were picked and re-streaked on the ISP2 agar plates supplemented with 30 μg/mL apramycin and then grown at 28 °C for 2 days. Colonies were inoculated into liquid YEME medium and rotationally incubated at 37 °C for 1 day to lose the temperature-sensitive plasmid. The cultures were then diluted and spread on plate. The colonies that are sensitive to apramycin were picked and subjected to DNA isolation and PCR validation. For further verification, two overlapping PCR fragments amplified with the primer pairs V-MshALOF/V-MshARIR and V-MshALIF/V-MshAROR were subjected to sequencing to make sure that no unintended mutations were introduced during the homologous recombination process (Supplementary Figure S18). The Δ*mst* mutant was generated in a similar way. For the complementation of *mshA*, a 1.4 kb DNA fragment containing the full-length coding sequence of *mshA* (*SVTN_RS20640*) and the putative ribosomal binding sequence was amplified by PCR with the primer pair Com-MshF/R. The amplified fragment was assembled into pSET-KasO* between *Afl*II and *Spe*I sites by using a ClonExpress kit. The resulting plasmid pSET-*mshA* was introduced into the Δ*mshA* mutant with the same method as described above. Screening of the desired complementary colony followed the standard procedure.

## Results

### Discovery of Sulfur-Containing Granaticin Congeners from *S*. *vietnamensis* GIMV4.0001

In our continuous studies on the granaticin biosynthesis, we noticed that there were two new minor peaks which possess characteristic UV absorptions of granaticins in some batches of fermentation in the liquid YEME medium. LC-MS analysis showed that the *m/z* values of the two minor peaks are 929.2501 [M–H]^–^ (Supplementary Figure S4) and 1043.3189 [M–H]^–^ (Supplementary Figure S5), respectively. Because the *m/z* values are different from the known granaticin congeners, we decided to isolate these compounds. During the isolation process, compound **3** came into our sights with a *m/z* value of 606.1285 [M–H]^–^ (Supplementary Figure S6) and granaticin-type UV absorption characteristics. Compound **3** couldn’t be detected by either HPLC or LC-MS in the samples of the direct fermentation broths or crude extracts of *S. vietnamensis*. It could only be detected after at least one round of silica gel separation. Therefore, compound **3** should be considered as a degradation product. Although the suggested molecular weight of compound **3** is as same as the compound 4-deoxy-4-S-(N-acetylcysteinyl) granaticinic acid (granaticin MA) which was reported nearly 40 years ago (Kormann et al., 1984), the NMR data and absolute configuration of granaticin MA were unavailable. It prompted us to purify it and collect the necessary spectrum data.

**FIGURE 2:**
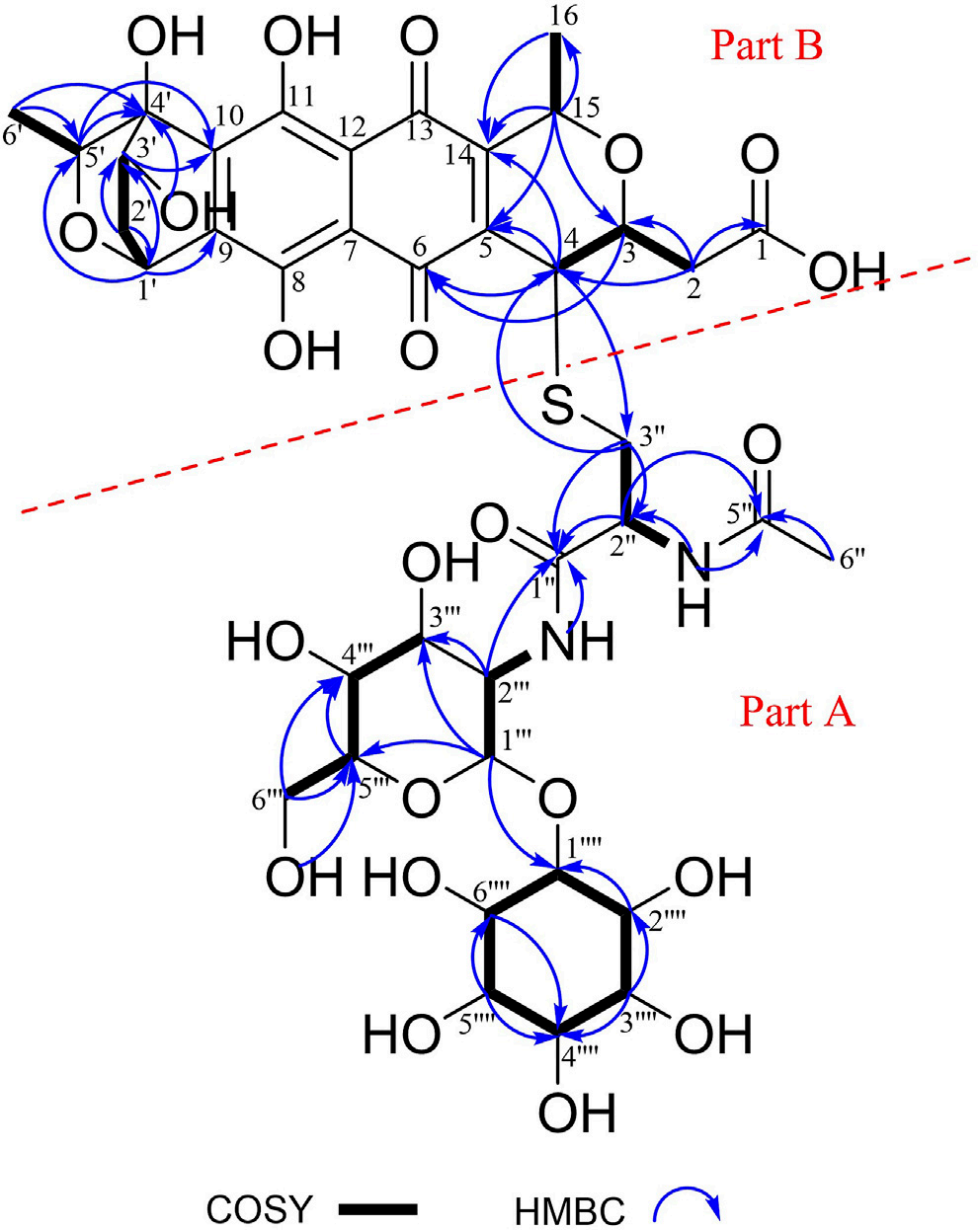
Key ^1^H–^1^H COSY and HMBC correlations of mycothiogranaticin A (**1**).

Compound **1** (2.06 mg/L) was obtained as a red powder [α]^25^_D_ -21.5 (*c* 0.016, CH_3_OH). Its molecular formula was established as C_39_H_50_N_2_O_22_S by HR-ESI-MS at *m/z* 929.2501 [M – H]^–^ (calcd. 929.2503), corresponding to sixteen degrees of unsaturation. The ^1^H and ^13^C NMR spectra (Supplementary Table S3), coupled with HSQC analysis, showed signals of three methyls, four methylenes, eighteen methines and fourteen quaternary carbons (Supplementary Figure S7–12). The signals of six methines [(*δ*_H_ 3.18, *δ*_C_ 80.2, C-1′′′′), (*δ*_H_ 3.91, *δ*_C_ 71.6, C-2′′′′), (*δ*_H_ 3.09, *δ*_C_ 71.6, C-3′′′′), (*δ*_H_ 3.34, *δ*_C_ 72.4, C-4′′′′), (*δ*_H_ 2.93, *δ*_C_ 74.8, C-5′′′′), (*δ*_H_ 3.53, *δ*_C_ 72.1, C-6′′′′)] were typical of an inositol moiety, further confirmed by the ^1^H–^1^H COSY correlations of H-1′′′′/H-2′′′′/H-3′′′′/H-4′′′′/H-5′′′′/H-6′′′′/H-1′′′′ (Figure 2). The signals of five methines [(*δ*_H_ 4.83, *δ*_C_ 98.6, C-1‴), (*δ*_H_ 3.64, *δ*_C_ 54.0, C-2‴), (*δ*_H_ 3.53, *δ*_C_ 70.9, C-3‴), (*δ*_H_ 3.10, *δ*_C_ 70.7, C-4‴), (*δ*_H_ 3.67, *δ*_C_ 72.9, C-5‴)] and one methylene (*δ*_H_ 3.44, *δ*_C_ 60.8, C-6‴) indicated the presence of an α-glucosamine moiety, which was verified by ^1^H–^1^H COSY correlations of H-1‴/H-2‴/NH-2‴ and H-3‴/H-4‴/H-5‴/H-6‴, along with the key HMBC correlations from H-1‴ to C-3‴ and C-5‴, and from H-2‴ to C-3‴. The ^1^H–^1^H COSY correlations of H-3′′/H-2′′/NH-2″ and the key HMBC correlations from H-2″ to C-1″ and C-5″, from NH-2″ and H-6″ to C-5″ confirmed the presence of an N-acetylcysteine moiety. Further analysis of the NMR data suggested that compound **1** contains a same moiety (part A in Figure 2) with nanaomycin H (Nakashima et al., 2017). The fragment could be verified by the key HMBC correlations from H-1‴ to C-1′′′′, from NH-2‴ and H-2‴ to C-1′′. Apart from the signals for the part A, the remaining signals (Supplementary Table S3) closely resembled those of dihydrogranaticin (Arnone et al., 1979), except for the presence of a methine group at C-4 of compound **1** instead of a methylene group in dihydrogranaticin. These findings suggested that part A and part B were linked through a sulfur atom between C-3″ and C-4. This deduction was confirmed by the HMBC correlations from H_2_-3″ to C-4 and from H-4 to C-3′′. Comprehensive analysis of ^1^H–^1^H COSY and HMBC spectra of compound **1** led to the establishment of its planar structure as depicted in Figure 2.

**FIGURE 3:**
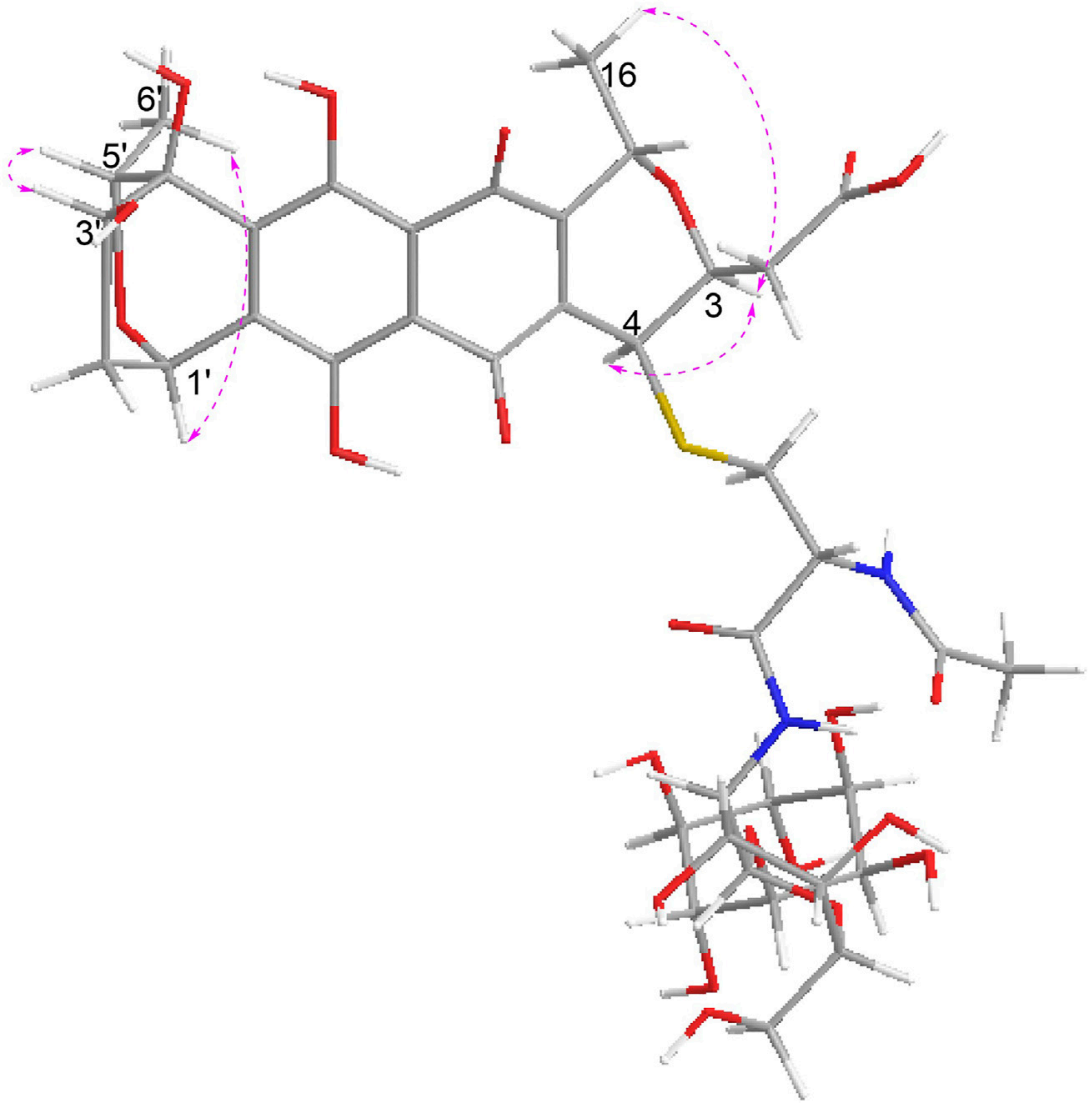
Key NOE correlations of mycothiogranaticin A (**1**).

**FIGURE 4:**
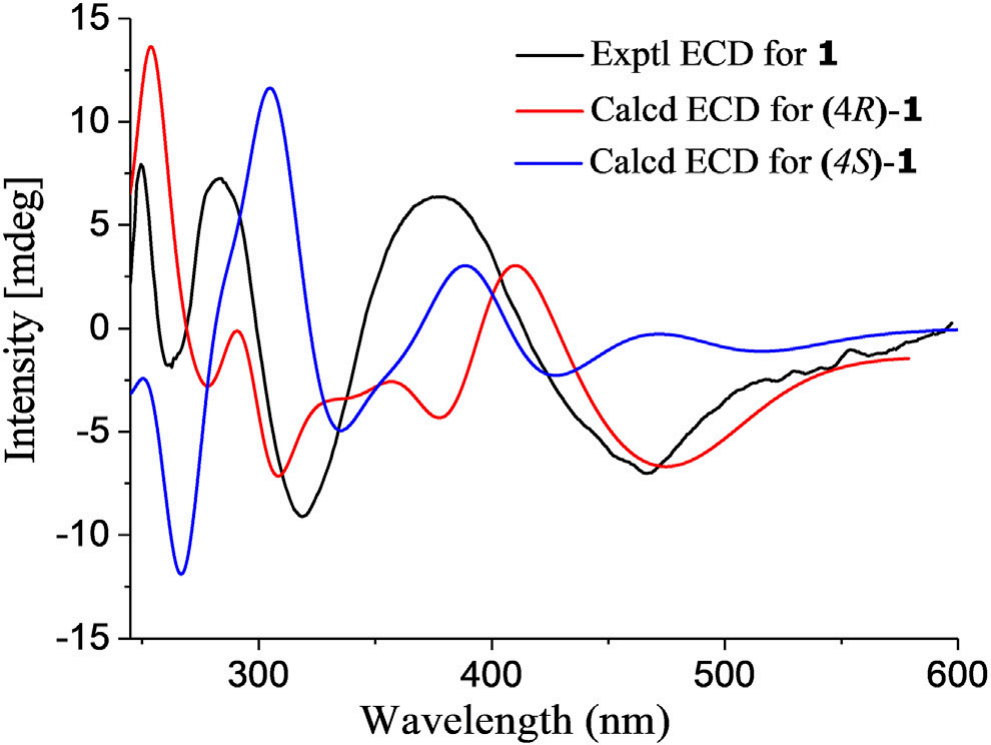
Experimental and calculated ECD spectra of mycothiogranaticin A (**1**).

In the NOESY spectrum (Figure 3), the observation of the NOE interactions of H-3/H_3_-16 revealed the same relative configuration of the right unit of part B of compound **1** to dihydrogranaticin. Meanwhile, the NOESY correlations of H-3′/H-5′ and H-1′/H-6′suggested the same relative configuration of the left unit of part B (Figure 1). Unfortunately, no NOESY correlations supported the relative configuration between the left and the right units of part B. However, the previously identified granaticin congeners from *S. vietnamensis* GIMV4.0001 have the same absolute configuration with the reported granaticins from *S. violaceoruber* Tü22 (Deng et al., 2011a). And the only biosynthetic gene cluster of granaticin residing in the genome of *S. vietnamensis* GIMV4.0001 shares identical organization and high sequence homology with that of *S. violaceoruber* Tü22. In view of biosynthesis, the absolute configuration of part B of compound **1** should be identical to that of dihydrogranaticin except for C-4. As for part A, till now, all the identified mycothiol S-conjugates share the same absolute configuration with mycothiol and the genetic engineering result further supported our conclusion that the part A of compound **1** share the same absolute configuration with mycothiol. Based on the above discussion, the absolute configuration of C-4 was determined as *R* by the NOESY correlation of H-3/H-4. This deduction was further verified by ECD/TDDFT computations on the two possible stereoisomers, (4*S*)-**1** and (4*R*)-**1**. As shown in Figure 4, the calculated ECD curve of (4*S*)-**1** showed the identical Cotton effects (CEs) as the experimental ECD curve for compound **1**. Consequently, the whole structure of compound **1** was established as shown in Figure 1, named as mycothiogranaticin A.

**FIGURE 5:**
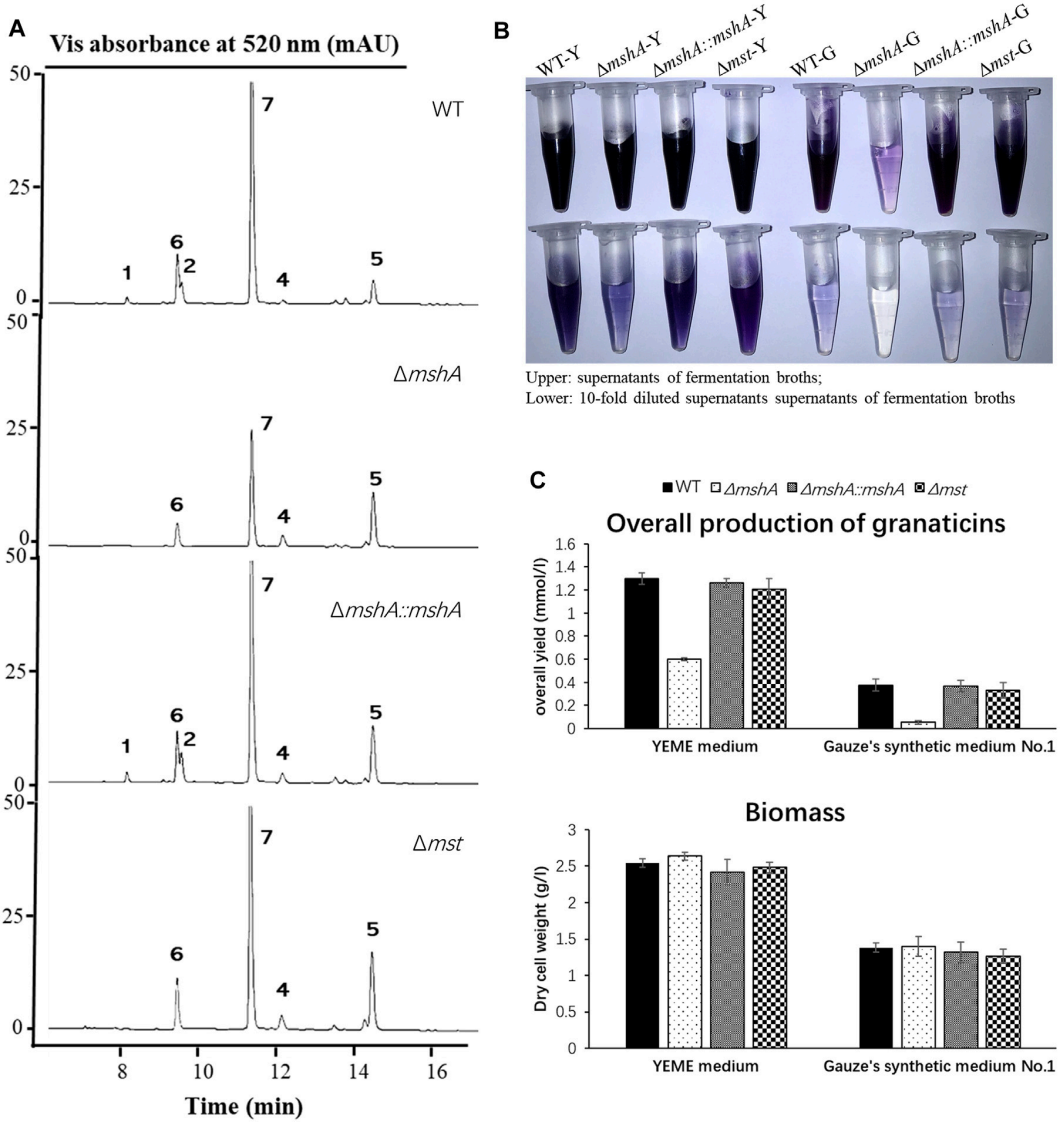
Effects of disruptions of the *mshA* and *mst* genes on the production of granaticins. **(A)** HPLC profiling of the *Streptomyces vietnamensis* wild type and genetically manipulated strains. Note that granaticin MA (**3**) might be a degradation product of mycothiogranaticin A (**1**) and could not be detected in the fermentation broth under the standard procedures. **(B)** The supernatants and diluted supernatants of the *S. vietnamensis* strains in different media. WT-Y, Δ*mshA*-Y, Δ*mshA*::*mshA*-Y, Δ*mst*-Y, WT-G, Δ*mshA*-G, Δ*mshA*::*mshA*-G and Δ*mst*-G stand for the wild-type (WT), mutant Δ*mshA*, complementary strain Δ*mshA*::*mshA* and mutant Δ*mst* in the YEME (Y) or Gauze’s synthetic No.1 (G) media, respectively. Tris-EDTA buffer (pH 8.0) was used for dilution. **(C)** The overall yields of granaticins and biomass of the wild type, mutant and complementary strains.

Compound **2** was prone to degrade during the purification process. Comparing the peaks of compounds **1** and **2** that showed in the HPLC profiles (Figure 5A), one would intuitively believe that the production titer of compound **2** should be greater than that of compound **1**. However, we regrettably did not obtain pure enough compound **2** to collect the NMR data. Compound **2** could turn into compound **1** rather rapidly, particularly at the stage of semi-preparation HPLC where 0.1% formic acid must be added into the mobile phase to enable the compounds to peak normally (Supplementary Figure S13). The molecular formula of compound **2** could be established as C_45_H_60_N_2_O_24_S with HR-ESI-MS data (exptl. *m/z* 1043.3189 [M – H]^–^; calcd. 1043.3184) (Supplementary Figure S14). When compound **2** was converted to compound **1**, the molecular weight was reduced by 114 Da, which is as same as in the situation of the conversion of granaticin B (**5**) into granaticin (**4**). The rhodinose moiety of granaticin B (**5**) is connected to the first sugar by an O-glycosidic bond, and this glycosidic bond is hypersensitive to acid and base. The reduction of 114 Da in molecular weight corresponded to the loss of the rhodinose moiety. This led us to speculate that compound **2** is a mycothiol S-conjugate of dihydrogranaticin B. Indeed, in the HRESI-MS/MS analysis, the major daughter peaks between compounds **2** and **1** shared either the same or a range of differences from 114.0672 to 114.0685 in *m/z* values (Supplementary Figure S15). This result is consistent with the expected conversion of the mycothiol S-conjugate of dihydrogranaticin B into the mycothiol S-conjugate of dihydrogranaticin. The MS/MS fragmentation mechanisms were proposed (Supplementary Figure S16). Thus, compound **2** was named as mycothiogranaticin B, and its proposed structure was shown in Figure 1.

Compound **3** (0.39 mg/L) was isolated as a red powder [α]^25^_D_ -654.0 (*c* 0.010, CH_3_OH). Its molecular formula was established as C_27_H_29_NO_13_S by HR-ESI-MS at *m/z* 606.1285 [M – H]^–^ (calcd. 606.1287). The 1D NMR spectroscopic data of compound **3** (Supplementary Table S4) were highly similar to those of compound **1**, except that the inositol and α-glucosamine moieties were absent and a carboxyl signal at C-1′′ (δ_C_ 173.8) was present. Further analyses of NMR data, including COSY and HMBC experiments (Supplementary Figure S17), elucidated the structure of compound **3** as 4-deoxy-4-S-(N-acetylcysteinyl) granaticinic acid (granaticin MA) (Kormann et al., 1984). Its absolute configuration was deduced as compound **1** by comprehensive consideration NMR data, biogenesis and genetic engineering result.

Mycothiogranaticin A (**1**) and granaticin MA (**3**) were tested for their potential antibacterial and cytotoxic activities. As shown in Supplementary Table S5, these two compounds, in comparison to granaticin (**4**), exhibited dramatically decreased activities against all the tested strains, and no inhibitory effects were observed against all the tested cancer cell lines (HL-60, MCF-7, HepG-2 and A549) and the LX-2 human hepatic stellate cell line (Supplementary Table S6).

### Gene Disruption Revealing Mycothiol as both a Structural Building Block and a Regulator in the Granaticin Biosynthesis

Mycothiol could be involved in antibiotic biosynthesis as exemplified by the biosynthesis of lincomycin A (Zhao et al., 2015). To investigate whether the biosynthesis of mycothiogranaticins is mycothiol pathway-dependent, we set out to generate an in-frame deletion mutant Δ*mshA*. The *mshA* gene was reported to be essential for mycothiol biosynthesis in *S. coelicolor* A3 (2) (Park et al., 2006) and other actinomycetes (Newton et al., 2003; Vilchèze et al., 2008). A BLAST analysis revealed that *SVTN_RS20640* is the best candidate gene of *mshA* (Supplementary Figure S2). Successful in-frame deletion of *mshA* (*SVTN_RS20640*) was verified by PCR confirmation of its genotype (Supplementary Figure S18). The mutant Δ*mshA* apparently produced much less blue pigment in the fermentation broth and no mycothiogranaticin A (**1**) or B (**2**) could be detected by HPLC analysis (Figures 5A, B). Reintroduction of *mshA* (*SVTN_RS20640*) into the mutant Δ*mshA* resulted in restoring the dark blue pigmentation of the fermentation broth and production of mycothiogranaticin A (**1**) and B (**2**) in the complementary strain Δ*mshA*::*mshA* (Figures 5A, B). The results suggested that the mycothiol moiety presented in mycothiogranaticins A (**1**) and B (**2**) is originated from the mycothiol pathway.

Mycothiol is a glutathione counterpart in many actinobacteria, and it is involved in cellular detoxification. The *mst* gene, encoding the mycothiol-S transferase, was reported to catalyze the conjugation of mycothiol to electrophiles to form mycothiol-electrophile conjugates (Newton et al., 2011). To further investigate the biosynthetic mechanism of mycothiogranaticins, *SVTN_RS22215*, the only *mst* candidate gene, was subjected to deletion. The genotype of the deletion mutant Δ*mst* (*SVTN_RS22215*) was confirmed by PCR and sequcencing (Supplementary Figure S19). Disruption of *SVTN_RS22215* would not affect the biosynthesis of both granaticin and mycothiol, but interrupt the conjugation step of mycothiol to granaticins. Indeed, the deletion mutant Δ*mst* didn’t show any reduced pigmentation of the fermentation broth (Figure 5B), but mycothiogranaticin A (**1**) and B (**2**) were absent from it (Figure 5A). The result showed that the mycothiol-S transferase is key for productions of mycothiogranaticins, suggesting that the biosynthesis of mycothiogranaticins relies on the mycothiol-dependent detoxification pathway, and that the incorporation of mycothiol into the granaticin chromophore should be the final step.

As mentioned earlier, disruption of *SVTN_RS20640* led much reduced pigmentation of the fermentation broth of the mutant strain Δ*mshA* (Figure 5B **)**, suggesting a negative effect on the granaticin production. The overall production of granaticins of the mutant strain Δ*mshA* were reduced by more than 50% in the YEME liquid and 80% in the Gauze’s synthetic medium No.1 liquid, respectively, while the complementary strain Δ*mshA*::*mshA* and the mutant strain Δ*mst* gave similar production levels to that of the wild type strain (Figure 5C). Theoretically, disruption of *mshA* (*SVTN_RS20640*) would result in mycothiol deficiency in the mutant strain Δ*mshA*. Considering the important role of mycothiol in maintaining the redox balance of the cytoplasm in *Actinobacteria* (Newton et al., 1996; Loi et al., 2015), the potential deleterious effect of the *mshA* deletion on cell growth and in turn reduction of the granaticin production should be examined. The growth of each strain was assessed by dry cell weighting. The result showed, however, the growth of the mutant strain Δ*mshA* was not impaired, comparing with that of the wild-type strain (Figure 5C). This suggested that the decreased production of granaticins in the mutant strain Δ*mshA* was correlated with the mycothiol deficiency. Mycothiol might possess a positive regulatory effect on the biosynthesis of granaticin.

## Discussion

A few of new granaticin congeners have been discovered in recent years (Jiang et al., 2014; Lv et al., 2019). Till now, the only sulfur-containing granaticin congener, granaticin MA, was reported nearly 4 decades ago (Kormann et al., 1984), but the NMR and bioactivity data were unavailable for the research community. In the current study, we discovered three granaticin congeners with mycothiol or N-acetylcysteine moieties, including granaticin MA, from *S. vietnamensis* GIMV4.0001. Sulfur incorporation can not only expand the structural diversity of molecules, but also endow them distinct bioactivities. For example, the S-bridged polyketide dimmer naquihexcin E possesses anti-HIV activity, whereas the antiviral activity of the non-sulfur-containing monomer naquihexcin K was not reported (He et al., 2019). More closely related examples are nanaomycin H, I and J (Nakashima et al., 2017; Matsuo et al., 2019a). Nanaomycin A and granaticin (**4**) are close BIQ members, and share the same stereo configuration (3*R*, 15*S*) in the pyran ring and only differ at the C-8 position of the lateral aromatic ring in the BIQ chromophores where the hydrogen atom in nanaomycin A is substituted by a hydroxyl group in granaticin (**4**). Like mycothiogranaticins, nanaomycin H, I and J contain mycothiol-derived moieties. While nanaomycin A shows strong antibacterial and cytotoxic activities, these sulfur-containing analogs showed, in contrast, no or weak antibacterial or anticancer bioactivities in the initial assay (Nakashima et al., 2017; Matsuo et al., 2019b). However, further bioactivity screening revealed that all these congeners possess epithelial-mesenchymal transition (EMT) inhibition activity showing potentials in invasive cancer therapy (Omura et al., 2018; Nakanishi et al., 2019). In spite of weak antibacterial activities and no cytotoxic activities revealed in the current study, mycothiogranaticin A (**1**) and granaticin MA (**3**) are still open for more bioactivity screening, particularly the evaluation of EMT inhibition activity, in consideration of the high similarity in structure between mycothiogranaticins and the mycothiol-derived nanaomycins.

**FIGURE 6:**
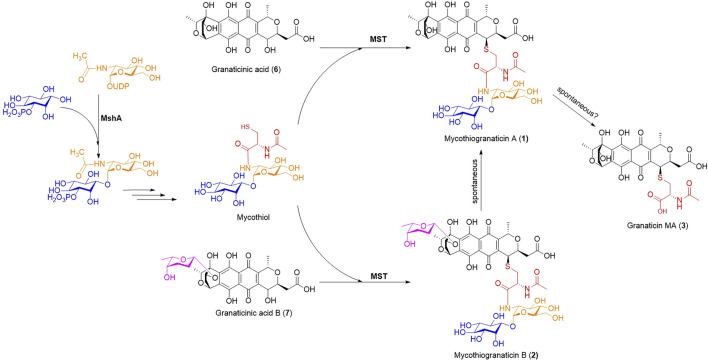
The proposed biosynthetic pathway of mycothiogranaticins. MshA, D-inositol-3-phosphate glycosyltransferase; MST, mycothiol-S transferase.

Although more and more sulfur-containing aromatic polyketides of bacterial origin, including sulfur-bridged dimers and mycothiol-derived monomers, have been discovered in recent years, the knowledge on the sulfur incorporation mechanism involved remains limited. Researchers proposed the recruitment of the mycothiol-dependent detoxification pathway for their biosynthesis (Wang et al., 2013; Fang et al., 2020). This assumption seems reasonable but has never been experimentally confirmed. A recent report showed that inorganic sulfur can be directly introduced into the polyketide chromophore to form sulfur-bridged dimers by nonenzymatic reactions (Cao et al., 2021), suggesting diverse sulfur incorporation mechanisms. In this study, we provided experimental evidence, for the first time, that mycothiogranaticins are derived from the mycothiol-dependent detoxification pathway. The proposed biosynthetic pathway was shown in Figure 6.

Mycothiol functions as a thiol-redox buffer to contribute to maintain the reduced state of the cytoplasm and mediates detoxification of both xenobiotic and endobiotic electrophilic compounds, resulting in mycothiol S-conjugates (Newton et al., 1996; Loi et al., 2015). Taking into account of the involvement of mycothiol in the biosynthesis and the reluctance of bioactivities, mycothiogranaticins should be recognized as detoxification products of granaticins by the producer. Surprisingly, disruption of *mshA* (*SVTN_RS20640*) reduced the granaticin production by at least more than 50%. Because reintroduction of *mshA* (*SVTN_RS20640*) into the Δ*mshA* mutant can restore the production level to that of the wild type, and disruption of *mst* (*SVTN_RS22215*) didn’t alter the overall yield of granaticins, mycothiol deficiency should account for the reduction in the Δ*mshA* mutant. This suggested that mycothiol is involved in positively tunning the production of granaticin. To the best of our knowledge, this is the first report that mycothiol can not only directly incorporate into polyketide structures, but also play an important regulatory role on polyketide biosynthesis.

Although we currently cannot figure out the underlying mechanism, the *soxR*-like *gra-orf20* gene, lying within the granaticin biosynthetic gene cluster, may serve as a clue for further investigation. In *E. coli*, the transcriptional factor SoxR, acting as a redox sensor system, governs a global defense against specific types of oxidative stress (Pomposiello and Demple, 2001). When oxidative stress occurs, the oxidized SoxR activates the transcription of the *soxS* gene which in turn activates the whole response regulon. In our previous study, disruption of *gra-orf20* unexpectedly led a three-fold increase of granaticin production, showing that this *soxR*-like gene played a negative regulatory role in the granaticin biosynthesis, probably by sensing the redox state of the cytoplasm, but the exact mechanism remains unknown (Deng M. et al., 2011). Because mycothiol is a major redox buffering agent in many actinomycetes, disruption of *mshA* (*SVTN_RS20640*) would result in mycothiol deficiency in the Δ*mshA* mutant and render this mutant more vulnerable to oxidative stress. In this situation, the SoxR homolog, Gra-ORF20, would be more easily and strongly activated, and in turn suppress the granaticin biosynthesis.

## Conclusion

Sulfur-containing polyketides of bacterial origin have received increasing attention from the fields of discovery and biosynthesis of natural products. In the current study, we discovered three sulfur-containing granaticin analogs, mycothiogranaticins A (**1**), B (**2**) and granaticin MA (**3**) from *S*. *vietnamensis* GIMV4.0001. The structure of mycothiogranaticin A (**1**) was determined by the comprehensive analysis of MS, NMR, ECD calculations and biosynthetic studies. The mycothiogranaticin A (**1**) and granaticin MA (**3**) showed dramatically decreased antibacterial activities and no cytotoxic activities. Gene disruptions suggested that the biosynthesis of mycothiogranaticins is mycothiol-dependent, providing experimental evidence, for the first time, for the biological origin of sulfur in this category of sulfur-containing polyketides. In addition, mycothiol was unexpectedly found to be involved in positive regulation of the biosynthesis of granaticins, probably by maintaining the cellular redox balance. To the best of our knowledge, this is the first report that mycothiol can not only be a building block of polyketides but also play a regulatory role in the polyketide biosynthesis.

## Data Availability

The original contributions presented in the study are included in the article/Supplementary Material, further inquiries can be directed to the corresponding authors.
