# Supplementary material for: Discovery of Mycothiogranaticins from Streptomyces vietnamensis GIMV4.0001 and the Regulatory Effect of Mycothiol on the Granaticin Biosynthesis
Source: Front Chem. 2021 Dec 23;9:802279. doi: 10.3389/fchem.2021.802279 (PMC8733708; doi:10.3389/fchem.2021.802279)
Supplement: Supplementary file 1 [file DataSheet1.docx]

Supplementary Material

**Supplementary Table S1.** Strains and plasmids used or generated in this study.

| **Strain/ plasmid** | **Description** | **Source (Reference)** |
| --- | --- | --- |
| **Strain** | | |
| *E. coli* | | |
| NEB turbo | *E. coli* host for general cloning | New England Biolabs |
| ET12567/pUZ8002 | Methylation-deficient *E. coli* host for intergeneric conjugation | (MacNeil et al., 1992) |
| *Streptomyces* | | |
| *Streptomyces vietnamensis* GIMV4.0001 | wild type strain | (Zhu et al., 2007) |
| Δ*mshA* (*SVTN_RS20640*) | in-frame deletion of *mshA* (*SVTN_RS20640*) | This study |
| Δ*mshA* (*SVTN_RS20640*)::*MshA* | *mshA*-complemented of the Δ*mshA* mutant | This study |
| Δ*mst* (*SVTN_RS22215*) | in-frame deletion of *mst* (*SVTN_RS22215*) | This study |
| **Plasmid** | | |
| pKC1139 | Apr^r^, temperature sensitive vector for conjugation | (Bierman et al., 1992) |
| pSET-KasO* | Apr^r^, pSET152 derived plasmid containing the promoter kasO* | (Pan et al., 2017) |
| pKC-Δ*mshA* | Apr^r^, in-frame deletion plasmid used for Δ*mshA* mutant construction | This study |
| pKC-Δ*mst* | Apr^r^, in-frame deletion plasmid used for Δ*mst* mutant construction | This study |
| pSET-*mshA* | Apr^r^, complemental plasmid used for re-introduction of *mshA* into the Δ*mshA* mutant | This study |

**Supplementary Table S2.** Primers used in this study.

| **Primer** | **Nucleotide Sequence (5'-3')** | **Description** |
| --- | --- | --- |
| Sv-MshALF | taaaacgacggccagtgccaagcTTCTTGCGGCCGTCCACGATC | Used for amplification of the homologous arms for the Δ*mshA* (*SVTN_RS20640*) mutant construction |
| Sv-MshALR | tcgccgttccGGGTGCCGGTGCCGGGTA |  |
| Sv-MshARF | catgtccgca CATGCACGACCACCGGCT |  |
| Sv-MshARR | acagctatgacatgattacgaattCGCGAGCACGGTGTTCTC |  |
| Sv-MstLF | taaaacgacggccagtgccaagcttCCGGATGTCTTCGTCCAC | Used for amplification of the homologous arms for the Δ*mst* (*SVTN_RS22215*) mutant construction |
| Sv-MstLR | gctttcccgcATCCGTACGGTGAGCTCC |  |
| Sv-MstRF | ccgtacggatGCGGGAAAGCTCCTGAAC |  |
| Sv-MstRR | acagctatgacatgattacgaattCCTCCAGCTGGTTGTAGATC |  |
| Com-MshF | gtgcaggactgggggagttcttaagTCCTTGAGTCGTGCCCGAGA | Used for construction of the complemental plasmid pSET-*mshA* |
| Com-MshR | aattcacgtcatatgtcagactagTCAGCCATGGTGCGAGCGTA |  |
| V-MshALOF | AAGAGCTGCGGGTCGTTCGT | Used for PCR confirmation of the genotype of the Δ*mshA* (*SVTN_RS20640*) mutant |
| V-MshARIR | TCAGCCATGGTGCGAGCGTA |  |
| V-MshALIF | TCCTTGAGTCGTGCCCGAGA |  |
| V-MshAROR | GACGTGTACAGCCCGCGGTC |  |
| V-MstLOF | AAGGGCTTCGGCTTCATC | Used for PCR confirmation of the genotype of the Δ*mst* (*SVTN_RS22215*) mutant |
| V-MstRIR | TGATCCCGTCATGCGAAC |  |
| V-MstLIF | TGTTGCTCGACTCGTTCGG |  |
| V-MstROR | GCCGGTGAGCTGCTTGTACG |  |

**Supplementary Table S3**. ^1^H NMR (700 MHz) and ^13^C NMR (175 MHz) data of mycothiogranaticin A (**1**) in DMSO-*d*_6_.

| Position | *δ*_C_, type | *δ*_H_ mult (*J* in Hz) |
| --- | --- | --- |
| 1 | 172.1, C |  |
| 2 | 37.9, CH_2_ | 2.74, dd (16.2, 8.8); 2.86, m |
| 3 | 66.9, CH | 4.41, m |
| 4 | 40.0, CH | 4.02, d (2.0) |
| 5 | 141.4, C |  |
| 6 | 181.3, C |  |
| 7 | 110.9, C |  |
| 8 | 154.3, C |  |
| 9 | 139.4, C |  |
| 10 | 137.4, C |  |
| 11 | 163.1, C |  |
| 12 | 110.6. C |  |
| 13 | 181.8, C |  |
| 14 | 144.0, C |  |
| 15 | 67.0, CH | 4.90, q (6.8) |
| 16 | 18.6, CH_3_ | 1.45, d (6.8) |
| 1' | 61.4, CH | 5.07, m |
| 2' | 37.1, CH_2_ | 1.31, d (14.1); 2.60, m |
| 3' | 70.3, CH | 3.84, m |
| 4' | 80.0, C |  |
| 5' | 71.7, CH | 3.68, m |
| 6' | 16.9, CH_3_ | 0.84, d (6.2) |
| 1'' | 170.0, C |  |
| 2'' | 52.9, CH | 4.43, m |
| 3'' | 34.6, CH_2_ | 2.85, m; 3.10, m |
| 5'' | 169.6, C |  |
| 6'' | 22.6, CH_3_ | 1.82, s |
| 1''' | 98.6, CH | 4.83, d (3.6) |
| 2''' | 54.0, CH | 3.64, m |
| 3''' | 70.9, CH | 3.53, m |
| 4''' | 70.7, CH | 3.10, m |
| 5''' | 72.9, CH | 3.67, m |
| 6''' | 60.8, CH_2_ | 3.44, m; 3.63, m |
| 1'''' | 80.2, CH | 3.18, m |
| 2'''' | 71.6, CH | 3.91, m |
| 3'''' | 71.6, CH | 3.09, m |
| 4'''' | 72.4, CH | 3.34, m, overlapped |
| 5'''' | 74.8, CH | 2.93, t (9.1) |
| 6'''' | 72.1, CH | 3.53, m |

**Supplementary Table S4**. ^1^H NMR (600 MHz) and ^13^C NMR (150 MHz) data of granaticin MA (**3**) in MeOD.

| Position | *δ*_C_, type | *δ*_H_ mult (*J* in Hz) |
| --- | --- | --- |
| 1 | 174.4, C |  |
| 2 | 39.1, CH_2_ | 2.91, m |
| 3 | 68.5 CH | 4.55, m |
| 4 | 41.7, CH | 4.12, d (1.8) |
| 5 | 142.8, C |  |
| 6 | 181.2, C |  |
| 7 | 112.5, C |  |
| 8 | 158.0, C |  |
| 9 | 141.6, C |  |
| 10 | 137.6, C |  |
| 11 | 163.8, C |  |
| 12 | 112.3. C |  |
| 13 | 182.3, C |  |
| 14 | 145.5, C |  |
| 15 | 69.2, CH | 5.02, q (6.6) |
| 16 | 19.1, CH_3_ | 1.56, d (6.6) |
| 1' | 63.4, CH | 5.20, dd (3, 1.8) |
| 2' | 37.9, CH_2_ | 1.44, d (15); 2.72, m |
| 3' | 72.2, CH | 3.96, dd (8.4, 1.8) |
| 4' | 81.4, C |  |
| 5' | 73.8, CH | 3.77, dd (12.6, 6) |
| 6' | 17.1, CH_3_ | 0.94, d (6) |
| 1'' | 173.8, C |  |
| 2'' | 54.2, CH | 4.67, dd (7.8, 4.8) |
| 3'' | 35.5, CH_2_ | 3.02, dd (13.2, 7.8); 3.22, dd(13.2, 4.2) |
| 5'' | 173.2, C |  |
| 6'' | 22.4, CH_3_ | 1.96, s |

**Supplementary Table S5.** Antibacterial activity of mycothiogranaticins A (**1**) and granaticin MA (**3**).

| Compound | MIC(µg/ml) | | |
| --- | --- | --- | --- |
|  | *Staphylococcus aureus*  ATCC 29213 | *Micrococcus luteus* GDMCC 1.932 | Methicillin-resistant *Staphylococcus aureus* ATCC 43300 |
| granaticin | 0.0625 | 0.5 | 0.0625 |
| mycothiogranaticin A | 32 | 32 | 64 |
| granaticin MA | 32 | 32 | 64 |
| Vancomycin*^a^* | 1 | 1 | 1 |

*^a^* Positive control.

**Supplementary Table S6.** Cytotoxicity of mycothiogranaticins A (**1**) and granaticin MA (**3**).

| Compounds | IC_50_*^a^* | | | | |
| --- | --- | --- | --- | --- | --- |
|  | SF-268 | MCF-7 | HepG-2 | A549 | LX-2 |
| Adriamycin*^b^* | 1.06±0.06 | 1.47±0.14 | 1.21±0.01 | 1.36±0.01 | 1.30±0.04 |
| Granaticin | 0.2±0.00 | 0.49±0.01 | 0.45±0.05 | 2.5±0.19 | 0.34±0.01 |
| mycothiogranaticin A | >128 | >128 | >128 | >128 | >128 |
| granaticin MA | >128 | >128 | >128 | >128 | >128 |

*^a^* Results are expressed as the mean IC_50_ ± SD values in μM. *^b^* Positive control.

**Supplementary Figure 1.** Standard calibration curves generated by using pure compounds for quantification of granaticins. The visible absorbance data were collected at 520 nm.


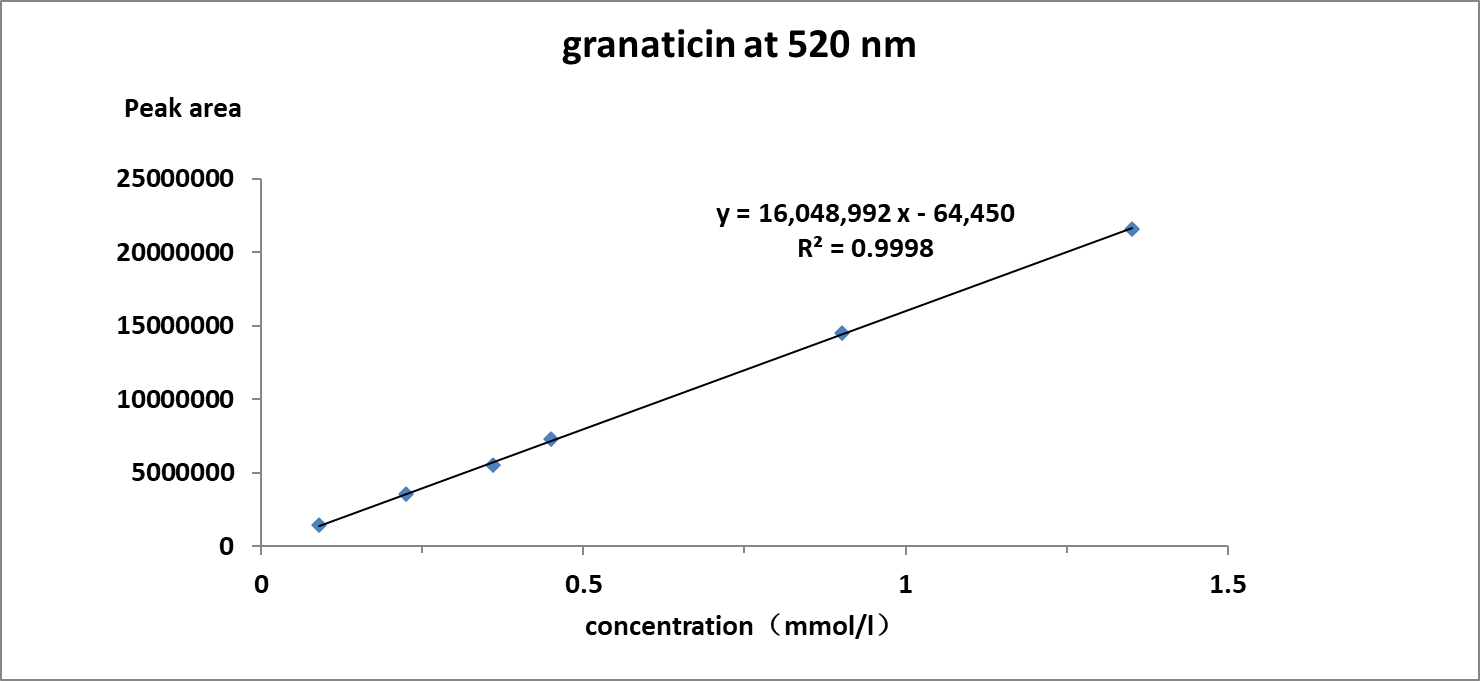

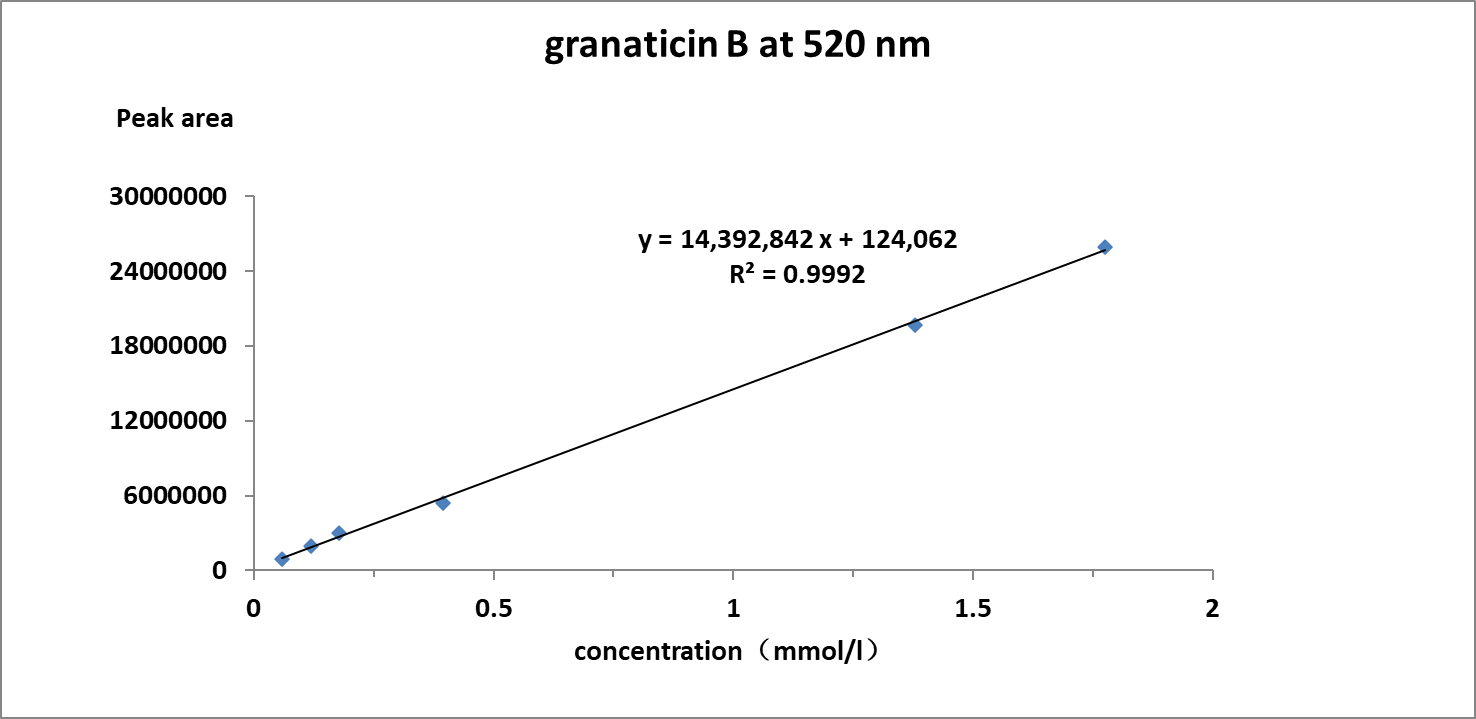


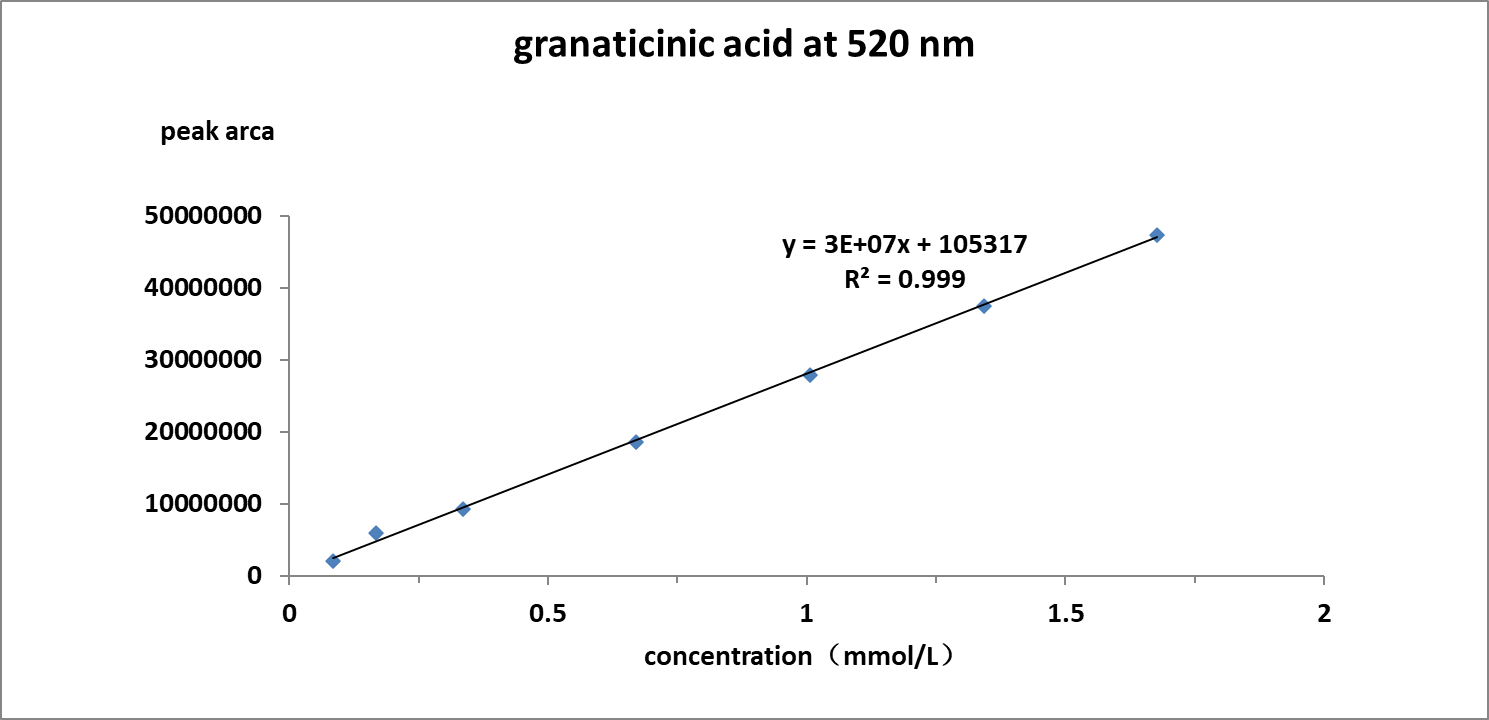

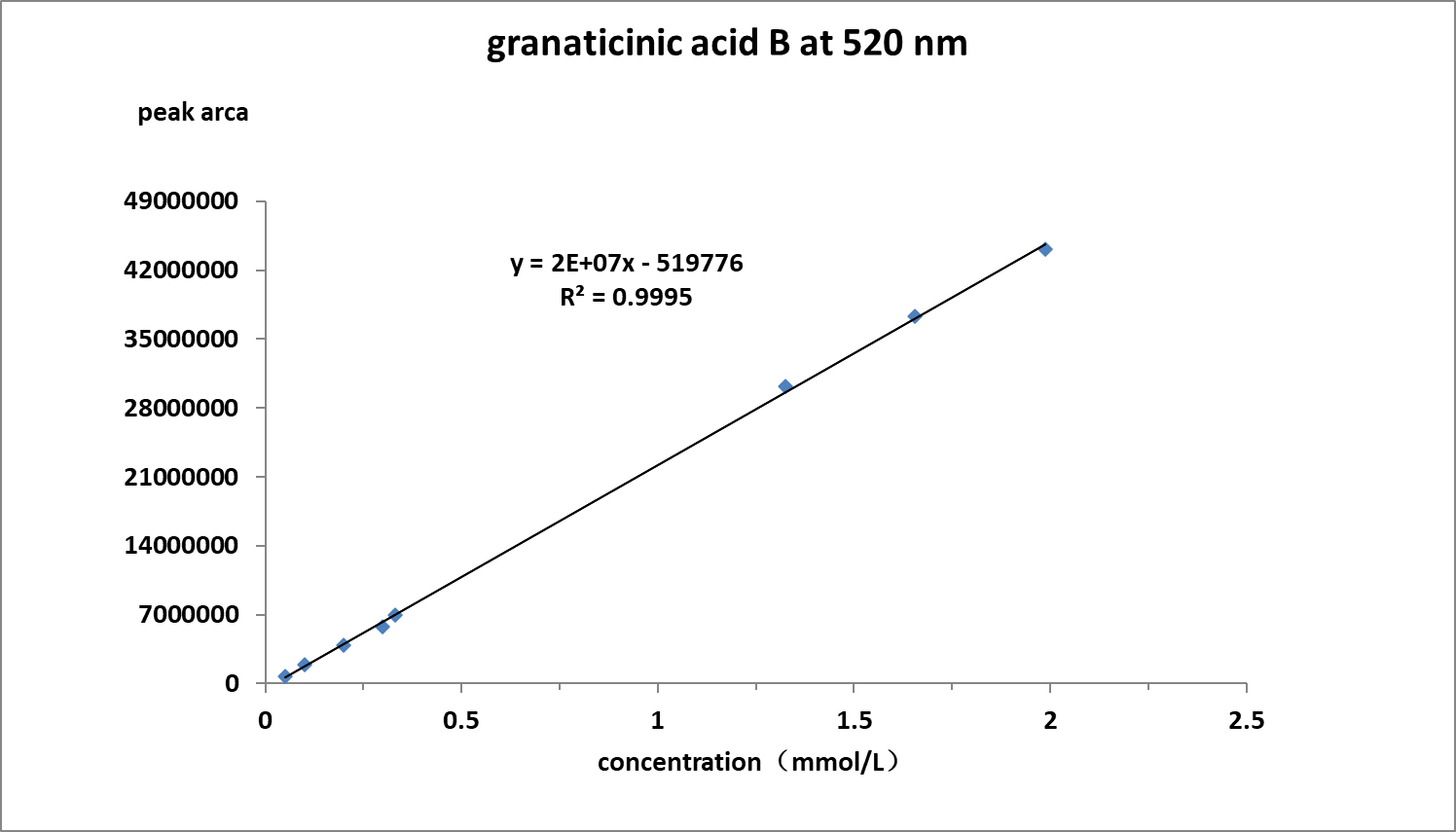


**Supplementary Figure 2.** GenBank BLAST result using the protein sequence of the D-inositol-3-phosphate glycosyltransferase (MshA, SCO4204) from *Streptomyces coelicolor* A3 (2) against the genome of *Streptomyces vietnamensis* GIMV4.0001. The sequence of WP_041130430.1 (encoded by *SVTN_RS20640*) shares an identity of as high as nearly 80%.


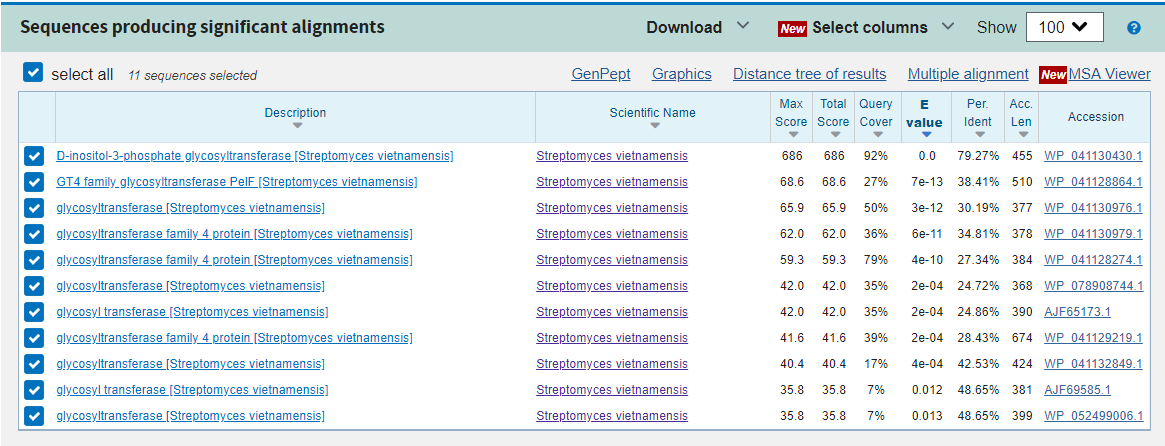


**Supplementary Figure 3.** GenBank BLAST result using the protein sequence of the mycothiol-S transferase (Mst, Rv0443) from *Mycobacterium tuberculosis* H37Rv against the genome of *Streptomyces vietnamensis* GIMV4.0001. The sequence of WP_041130676.1 (encoded by *SVTN_RS22215*) shares an identity of 52.8%.


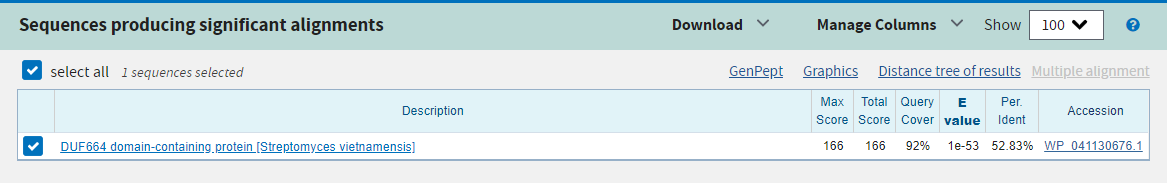


**Supplementary Figure 4.** HRESIMS spectrum of mycothiogranaticin A (**1**).

**Supplementary Figure 5.** HRESIMS spectrum of compound **2**.

**Supplementary Figure 6.** HRESIMS spectrum of 4-deoxy-4-S-(N-acetylcysteinyl) granaticinic acid (granaticin MA, **3**).

**Supplementary Figure 7.** ^1^H NMR spectrum of mycothiogranaticin A (**1**) in DMSO-*d*_6_.

**
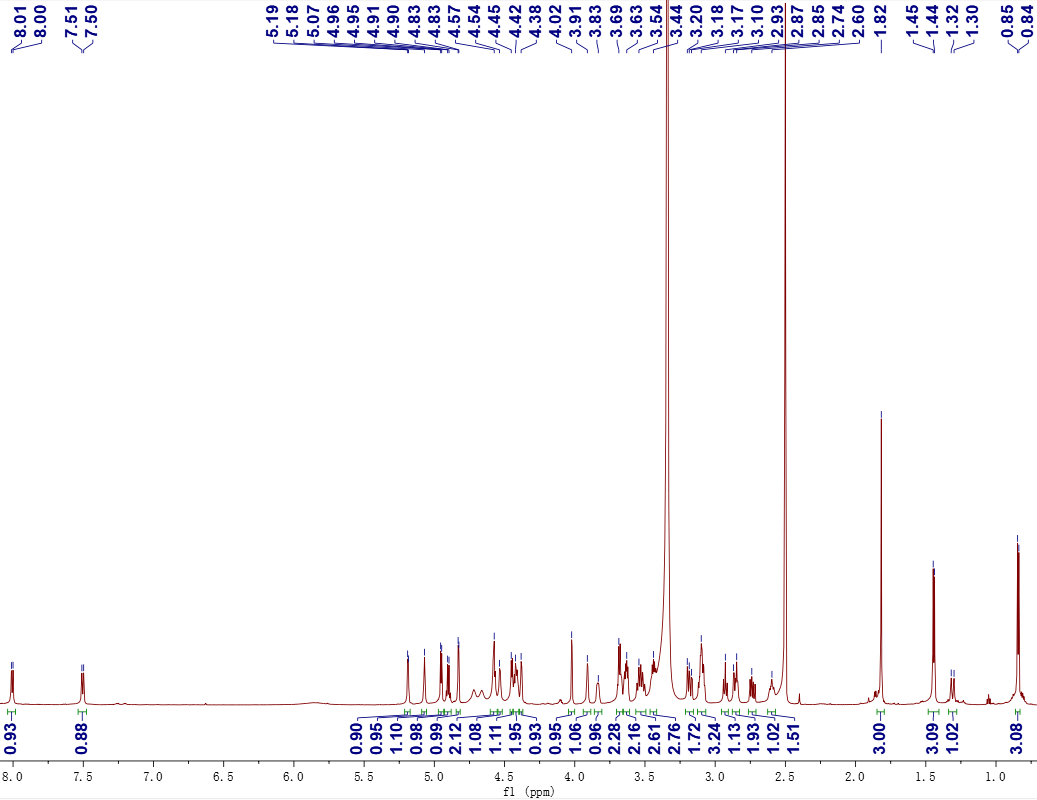
**

**Supplementary Figure 8.** ^13^C NMR spectrum of mycothiogranaticin A (**1**) in DMSO-*d*_6_.

**
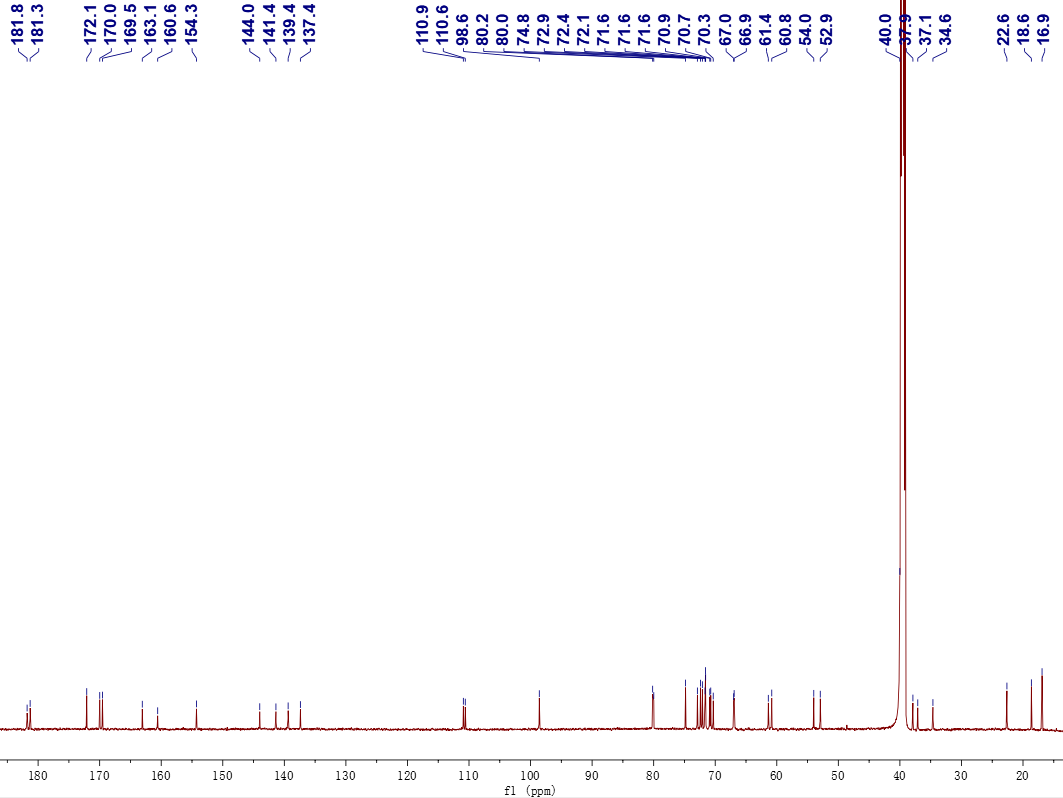
**

**Supplementary Figure 9.** HSQC spectrum of mycothiogranaticin A (**1**) in DMSO-*d*_6_.

**
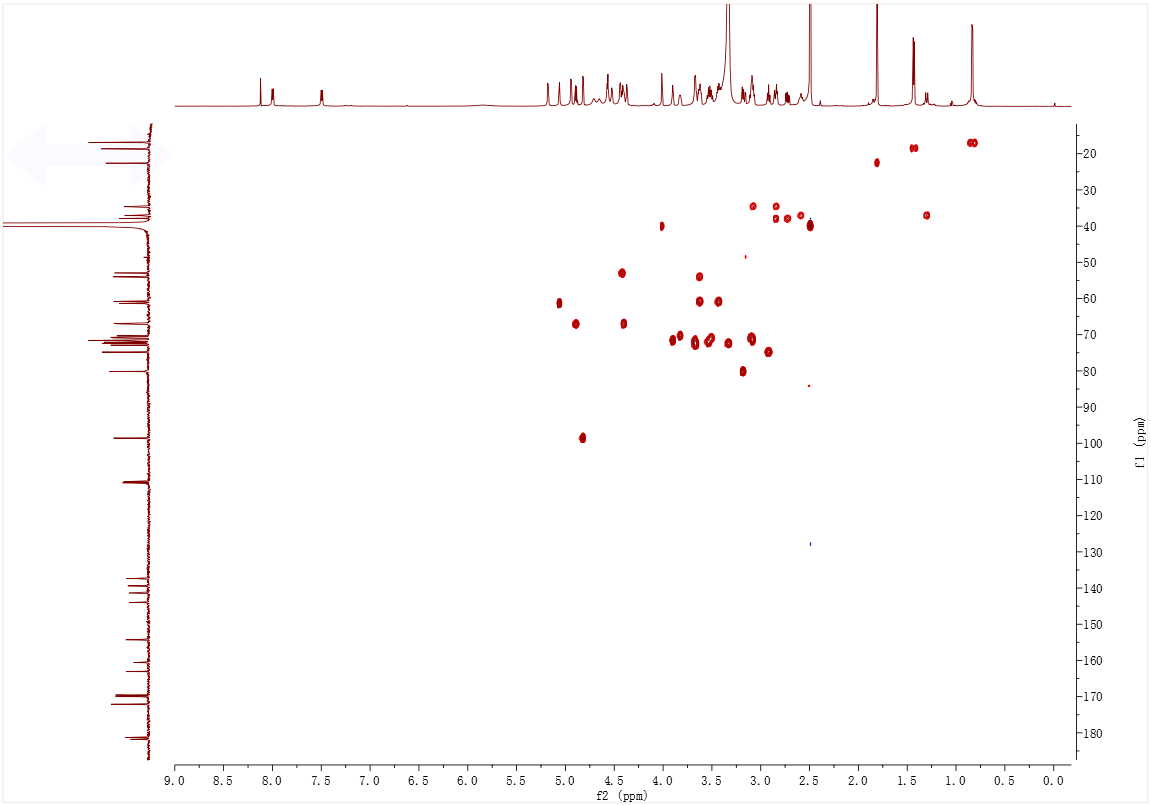
**

**Supplementary Figure 10.** ^1^H-^1^H COSY spectrum of mycothiogranaticin A (**1**) in DMSO-*d*_6_.

**
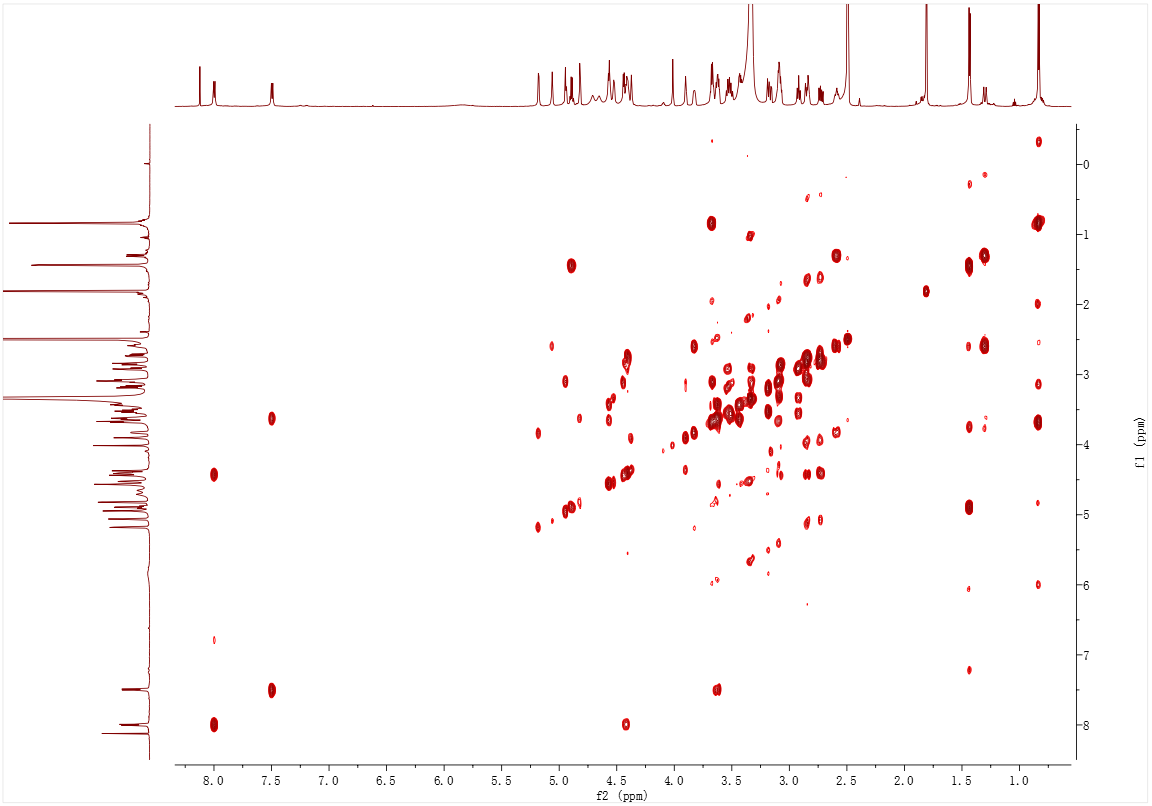
**

**Supplementary Figure 11.** HMBC spectrum of mycothiogranaticin A (**1**) in DMSO-*d*_6_.

**
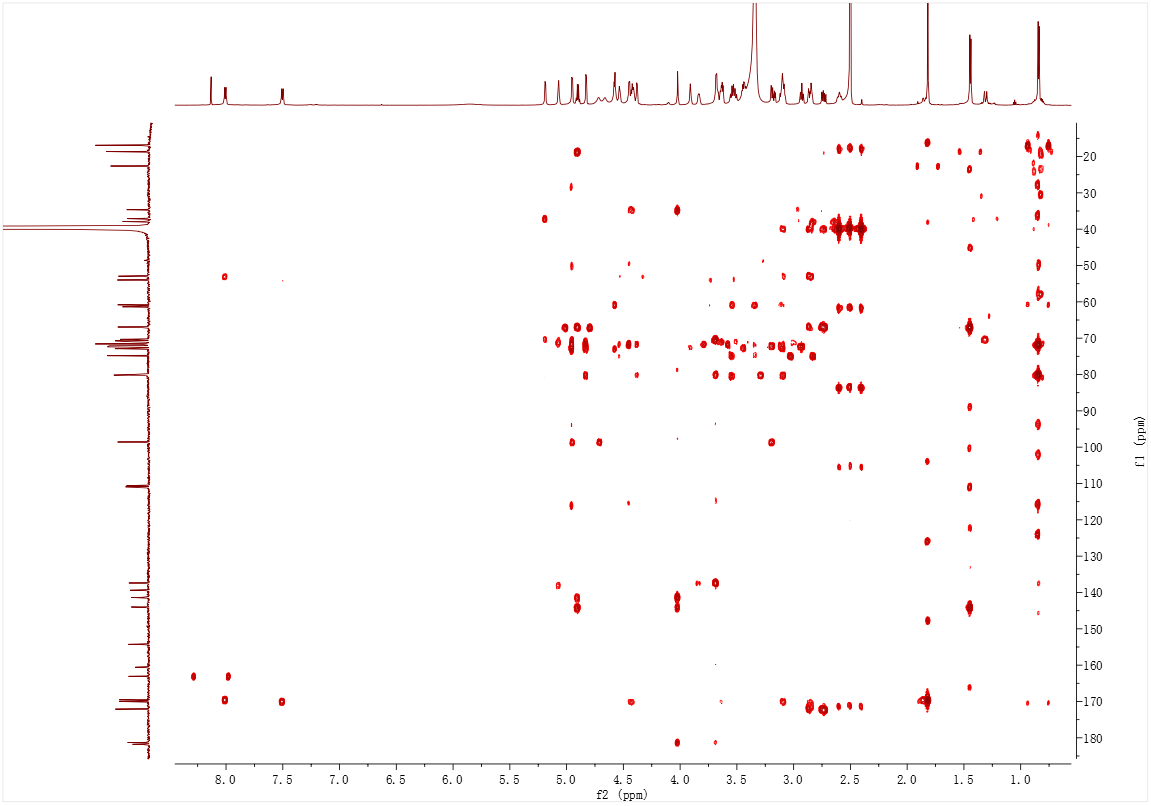
**

**Supplementary Figure 12.** NOESY spectrum of mycothiogranaticin A (**1**) in DMSO-*d*_6_.

**
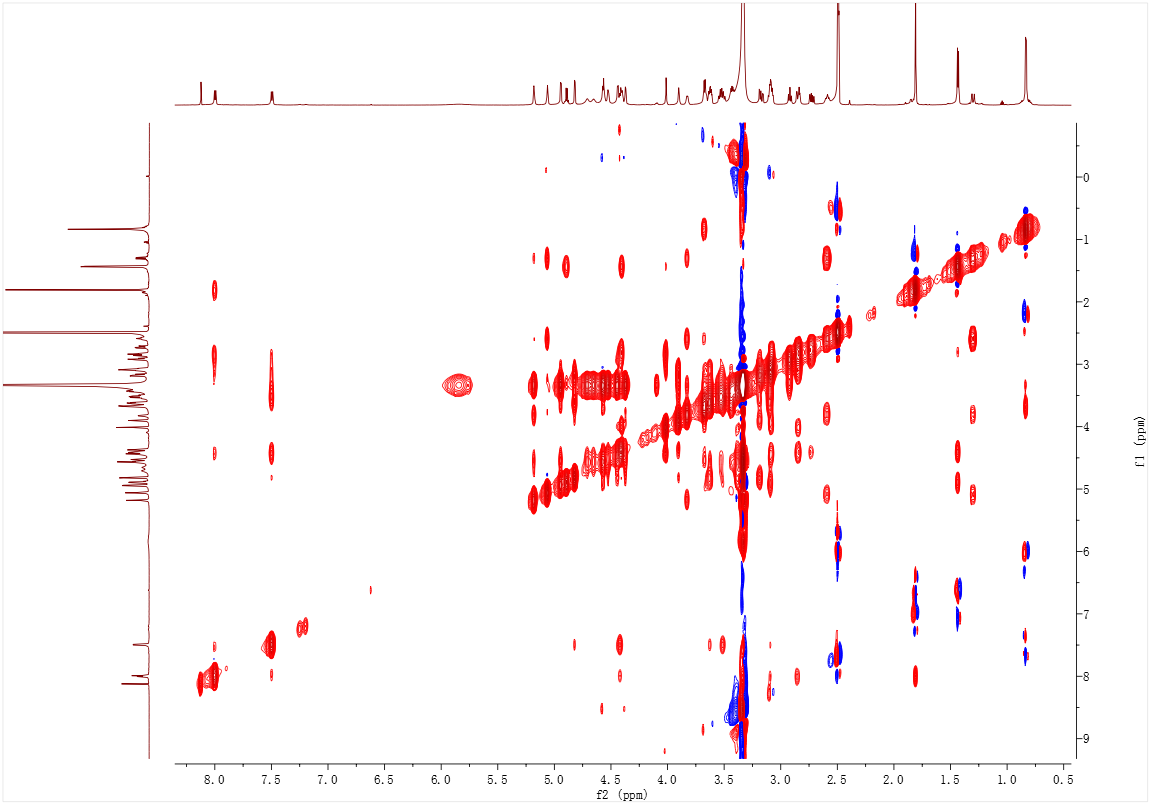
**

**Supplementary Figure 13.** Compound **2** spontaneously degraded into compound **1** in the purification process.

**
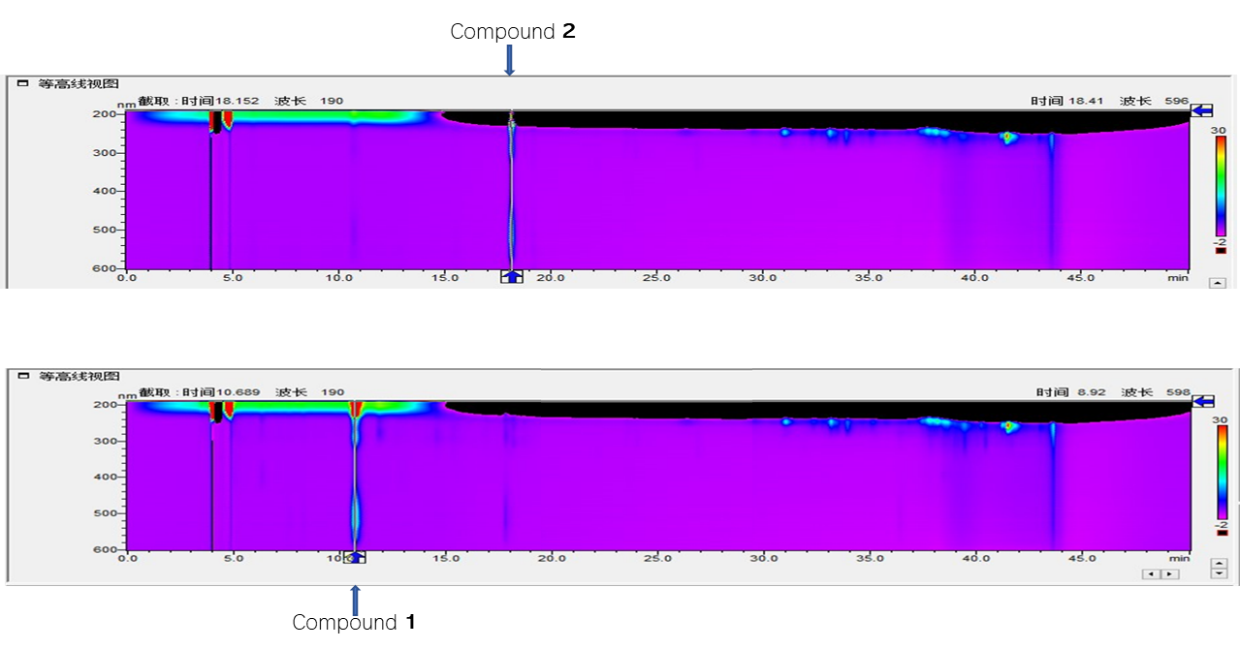
**

**Supplementary Figure 14.** Predicted chemical formula of compound **2** by *m/z* value.


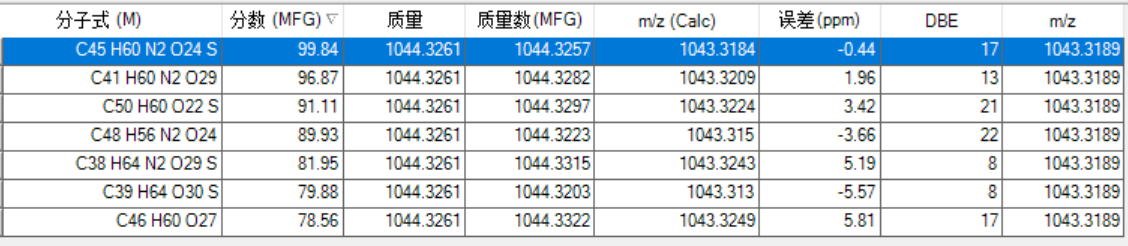


**Supplementary Figure 15.** Comparison of HRESI-MS/MS spectra of compounds **1** and **2**.

Mycothiogranaticin A (**1**)

Mycothiogranaticin B (**2**)

**Supplementary Figure 16.** Proposed MS/MS fragmentation mechanisms of compounds **1** (A) and **2** (B) in negative mode.


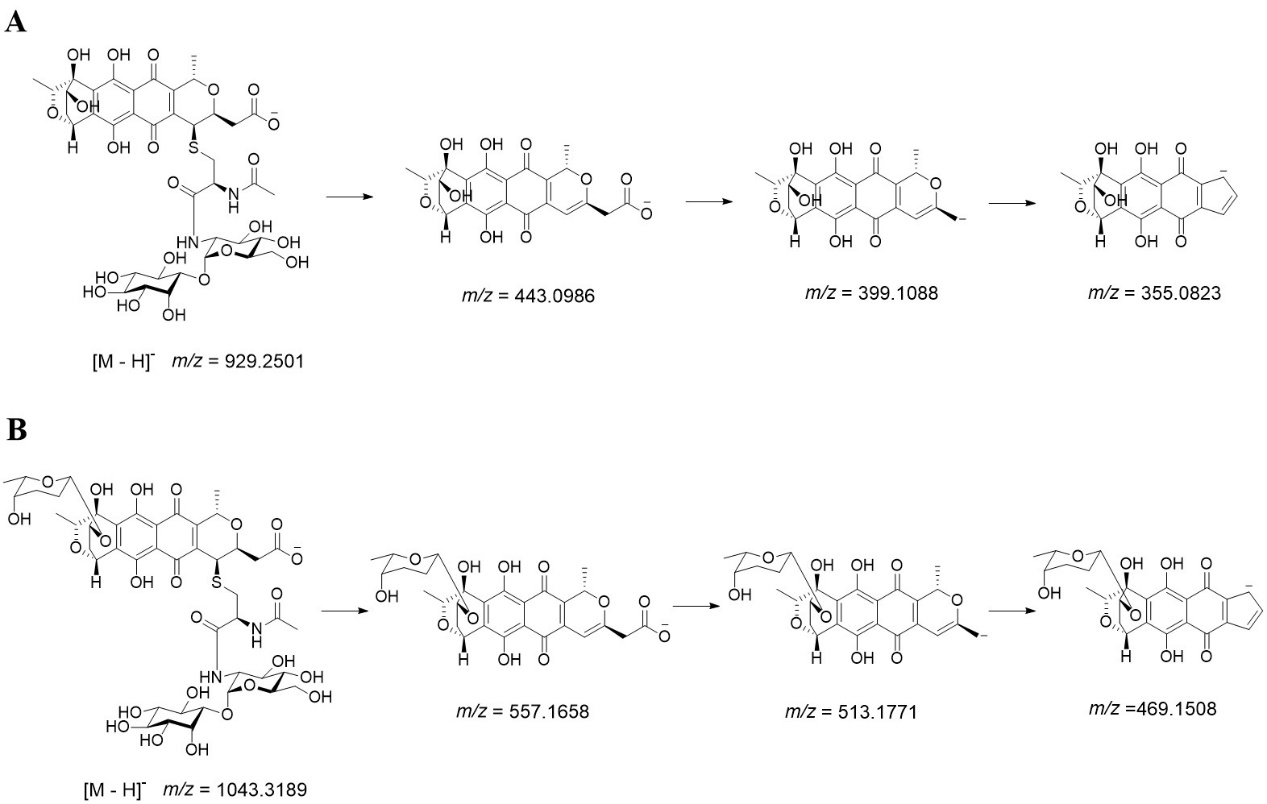


**Supplementary Figure 17.** Key ^1^H–^1^H COSY and HMBC correlations of 4-deoxy-4-S-(N-acetylcysteinyl) granaticinic acid (granaticin MA, **3**).

**Supplementary Figure 18.** Construction of the in-frame deletion mutant strain Δ*mshA* (*SVTN_RS20640*) and confirmation of its genotype by PCR analysis. (**A**) Schematic representation for the deletion of Δ*mshA* (*SVTN_RS20640*) in the wild-type strain of *Streptomyces vietnamensis* GIMV4.0001 by homologous recombination. Three sets of primers were used to verify the expected in frame deletion. The fragments amplified with V-MshALOF/V-MshARIR, V-MshALIF/V-MshAROR or V-MshALIF/V-MshARIR from the wild-type strain GIMV4.0001 are 3204, 3111 and 11435 -bp long, respectively. Those from the mutant strain [Δ*mshA* (*SVTN_RS20640*)] are 1941, 1848 and 172 -bp long, respectively. Primers V-MshALOF and V-MshAROR stand outside of the homologous arms. (**B**) PCR verification of the in-frame deletion mutant strain Δ*mshA* (*SVTN_RS20640*). w, wild-type; m, mutant. Lanes 1 and 8, DNA marker DL5000; lanes 2 and 3, products amplified with V-MshALOF/V-MshARIR; lanes 4 and 5, products amplified with V-MshALIF/V-MshAROR; lanes 6 and 7, products amplified with V-MshALIF/V-MshARIR.


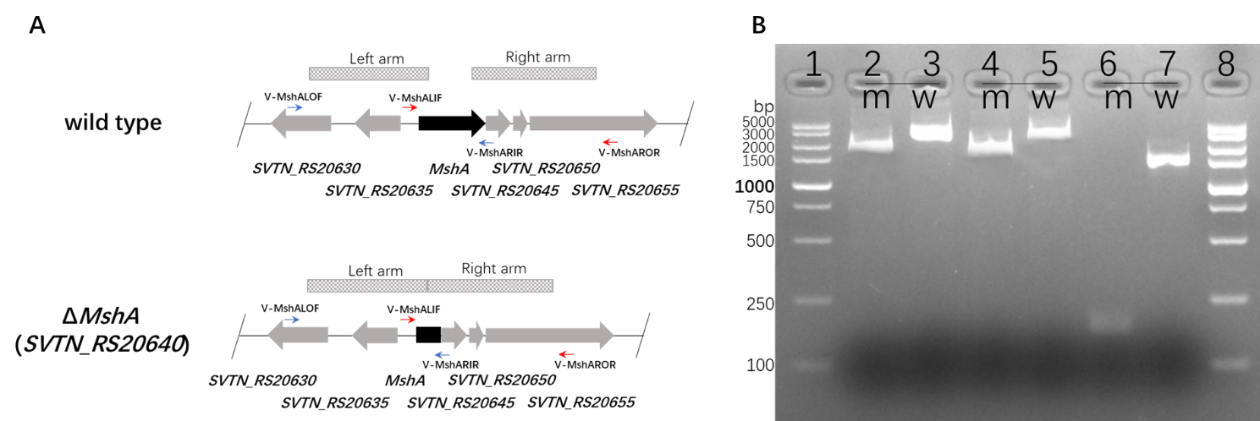


**Supplementary Figure 19.** Construction of the in-frame deletion mutant strain Δ*mst* (*SVTN_RS22215*) and confirmation of its genotype by PCR analysis. (A) Schematic representation for the deletion of Δ*mst* (*SVTN_RS22215*) in the wild type strain of *Streptomyces vietnamensis* (GIMV4.0001) by homologous recombination. Three sets of primers were used to verify the expected in frame deletion. The fragments amplified with V-MstLOF/V-MstRIR, V-MstLIF/V-MstROR or V-MstLIF/V-MstRIR from the wild type strain (GIMV4.0001) are 2289, 2455 and 533 -bp long, respectively. Those from the mutant strains [Δ*mst* (*SVTN_ RS22215*)] are 1890, 2056 and 134 -bp long, respectively. Primers V-MstLOF and V-MstROR stand outside of the homologous arms. (B) PCR verification of the in-frame deletion mutant strain Δ*mst* (*SVTN_ RS22215*). w, wild type; m, mutant. Lanes 1 and 11, DNA marker DL5000; lanes 2 to 4, products amplified with V-MstLOF/V-MstRIR; lanes 5 to 7, products amplified with V-MstLIF/V-MstROR; lanes 8 to 10, products amplified with V-MstLIF/V-MstRIR.

**
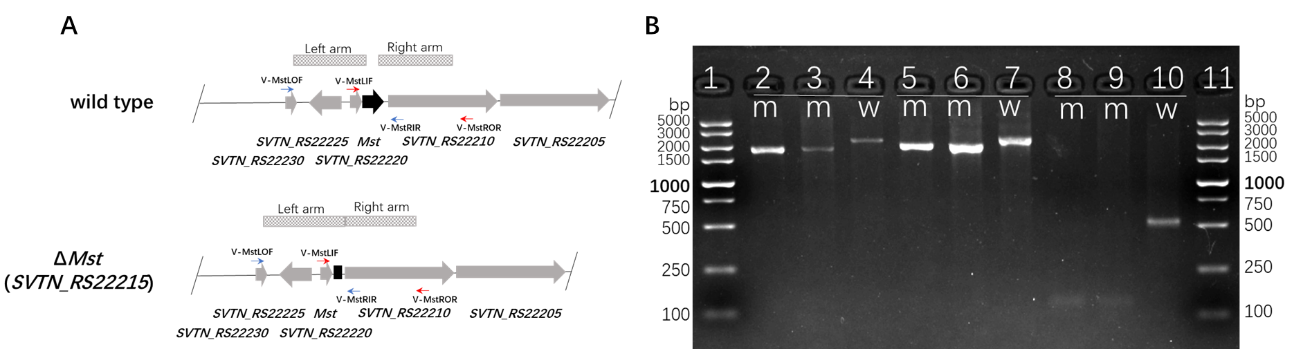
**

**Reference**

Bierman, M., Logan, R., O'Brien, K., Seno, E.T., Nagaraja Rao, R., and Schoner, B.E. (1992). Plasmid cloning vectors for the conjugal transfer of DNA from *Escherichia coli* to *Streptomyces* spp. *Gene* 116(1)**,** 43-49. doi: https://doi.org/10.1016/0378-1119(92)90627-2.

MacNeil, D.J., Gewain, K.M., Ruby, C.L., Dezeny, G., Gibbons, P.H., and MacNeil, T. (1992). Analysis of *Streptomyces avermitilis* genes required for avermectin biosynthesis utilizing a novel integration vector. *Gene* 111(1)**,** 61-68. doi: 10.1016/0378-1119(92)90603-M

Pan, G., Xu, Z., Guo, Z., Hindra, Ma, M., Yang, D., et al. (2017). Discovery of the leinamycin family of natural products by mining actinobacterial genomes. *Proceedings of the National Academy of Sciences* 114(52)**,** E11131-E11140. doi: 10.1073/pnas.1716245115.

Zhu, H.-h., Guo, J., Yao, Q., Yang, S.-z., Deng, M.-r., Phuong, L.T.B., et al. (2007). *Streptomyces vietnamensis* sp. nov., a streptomycete with violet blue diffusible pigment isolated from soil in Vietnam. *International Journal of Systematic and Evolutionary Microbiology* 57(8)**,** 1770-1774. doi: 10.1099/ijs.0.64774-0.
